# Differential Modulation of GABAergic and Glutamatergic Neurons in the Ventral Pallidum by GABA and Neuropeptides

**DOI:** 10.1523/ENEURO.0404-22.2023

**Published:** 2023-07-10

**Authors:** Daniela Neuhofer, Peter Kalivas

**Affiliations:** Department of Neurosciences, Medical University of South Carolina, Charleston, South Carolina 29425

**Keywords:** GABA, medium spiny neurons, neuropeptides, optogenetics, ventral pallidumg, whole cell patch clamp

## Abstract

The ventral pallidum (VP) is an integral locus in the reward circuitry and a major target of GABAergic innervation of both D1-medium spiny neurons (MSNs) and D2-MSNs from the nucleus accumbens. The VP contains populations of GABAergic [VPGABA, GAD2(+), or VGluT(–)] and glutamatergic [VPGlutamate, GAD2(–), or VGluT(+)] cells that facilitate positive reinforcement and behavioral avoidance, respectively. MSN efferents to the VP exert opponent control over behavioral reinforcement with activation of D1-MSN afferents promoting and D2-MSN afferents inhibiting reward seeking. How this afferent-specific and cell type-specific control of reward seeking is integrated remains largely unknown. In addition to GABA, D1-MSNs corelease substance P to stimulate neurokinin 1 receptors (NK1Rs) and D2-MSNs corelease enkephalin to activate μ-opioid receptors (MORs) and δ-opioid receptors. These neuropeptides act in the VP to alter appetitive behavior and reward seeking. Using a combination of optogenetics and patch-clamp electrophysiology in mice, we found that GAD2(–) cells receive weaker GABA input from D1-MSN, but GAD2(+) cells receive comparable GABAergic input from both afferent types. Pharmacological activation of MORs induced an equally strong presynaptic inhibition of GABA and glutamate transmission on both cell types. Interestingly, MOR activation hyperpolarized VPGABA but not VGluT(+). NK1R activation inhibited glutamatergic transmission only on VGluT(+) cells. Our results indicate that the afferent-specific release of GABA and neuropeptides from D1-MSNs and D2-MSNs can differentially influence VP neuronal subtypes.

## Significance Statement

Little is known about the cell type-specific modulation of neurotransmission by the neuropeptides coreleased with GABA from striatal D1 and D2 synapses. We explored the differential microcircuitry of the D1 and D2 inputs to the GABAergic and glutamatergic neuronal subpopulations in the ventral pallidum. Based on differential electrophysiological actions of D1 and D2 GABAergic and peptidergic inputs, we propose a circuit-based model that partly explains the different actions of D1-MSN and D2-MSN afferents in the ventral pallidum to regulate reward behaviors.

## Introduction

The ventral pallidum (VP) is an integral component of the reward circuitry that translates motivation into action (Kalivas et al., 1999; Smith et al., 2009). In addition to strong GABAergic innervation from the nucleus accumbens (NAc), the VP also receives glutamatergic innervation from the basolateral amygdala and subthalamic nucleus, cholinergic innervation from local interneurons, as well as dopaminergic innervation from the ventral tegmental area (VTA; Root et al., 2015). VP neurons encode several integral components of reward processing and behavioral execution such as the valence of reward (Tindell et al., 2005; Ottenheimer et al., 2018), reward prediction errors (Ottenheimer et al., 2020; Stephenson-Jones et al., 2020), and the initiation of reward-seeking behavior (Richard et al., 2016).

At a cellular level, the VP is composed of heterogeneous subpopulations of neurons that differentially use GABA (VPGABA; ∼70%) or glutamate (VPGlutamate; ∼25%) as neurotransmitters (Heinsbroek et al., 2020). Both cell types drive opponent behaviors, with activation of VPGABA neurons supporting the seeking of positive reinforcement and activation of VPGlutamate facilitating behavioral avoidance (Faget et al., 2018; Tooley et al., 2018; Heinsbroek et al., 2020; Stephenson-Jones et al., 2020). Additionally, stress-induced adaptations in these two subpopulations of VP neurons differentially contribute to behavioral despair and social withdrawal, two key symptoms of depressive disorder (Knowland et al., 2017).

Both D1-medium spiny neurons (MSNs) and D2-MSNs from the NAc form functional synapses in the VP and exert opponent control on reward-related behaviors (Kupchik et al., 2015; Creed et al., 2016). Chemogenetic manipulations demonstrate that D1-MSN to VP afferents promote and D2-MSN to VP afferents inhibit reward seeking (Creed et al., 2016; Heinsbroek et al., 2017; Pardo-Garcia et al., 2019). How this antagonistic behavioral modulation is achieved on a microcircuit level in the VP remains unclear. However, a rabies tracing study revealed that significantly more D1-MSNs than D2-MSNs in the NAc innervate VPGlutamate cells (Heinsbroek et al., 2020). Nonetheless, it remains unknown whether the apparently biased connectivity between NAc afferents and VP neuronal subpopulations is reflected in GABA synaptic strength and modulation by cotransmitters differentially released with GABA from D1-MSN and D2-MSN axon terminals.

D1-MSNs release the neuropeptides substance P and dynorphin [activating neurokinin 1 receptors (NK1Rs) and κ opioid receptors, respectively], and D2-MSNs release enkephalin and neurotensin [activating μ-opioid receptors (MORs) and NK1Rs, respectively; Smith et al., 2013]. These neuropeptides modulate excitatory and inhibitory synaptic transmission in the NAc (Tejeda et al., 2017; Francis et al., 2019) and regulate motivated behaviors in the VP (Hasenöhrl et al., 2000; Tang et al., 2005; Torregrossa and Kalivas, 2008). Hence, distinct neuropeptides released from D1 and D2 terminals could gate VP output in a cell type-specific way and allow for fine-tuned control of motivated behaviors.

Here we used circuit-specific optogenetics in transgenic mouse lines expressing a reporter in VPGABA neurons [VPGAD(+)] combined with whole-cell patch-clamp recordings to characterize the functional connectivity between the D1/D2-MSNs and VPGABA/VPGAD2(–) neurons and to test an hypothesis that the apparent bias in anatomic connectivity (Heinsbroek et al., 2020) is recapitulated in biased functional connectivity. We furthermore used MOR and NK1R agonists to evaluate the potential cell type-specific modulation by two of the coreleased neuropeptides. Given the previous literature that the stimulation of MOR or NK1R in the VP can promote motivated behavior, we hypothesized that agonists at these receptors would either stimulate VPGABA or inhibit VPGlutamate.

We found that while VPGABA cells receive comparable input from D1-MSN and D2-MSN afferents, VPGAD2(–) receives significantly lower GABA_A_ receptor (GABA_A_R)-mediated transmission from D1-MSNs compared with D2-MSNs. MOR activation inhibited both GABA and glutamate presynaptic transmission onto both cell types to a similar extent. However, MOR stimulation had a postsynaptic effect (hyperpolarization and reduced firing) only in VPGAD2(+) but not VPGAD2(–). NK1R activation inhibited glutamatergic transmission only onto VPGAD2(–) cells. Hence, the neuropeptidergic control via the D2 coreleased neuropeptide enkephalin more tightly controlled VPGABA neurons, and the D1 coreleased neuropeptide only controls synaptic input onto VPGlutamate neurons.

## Materials and Methods

### Animals

Male and female transgenic mice [postnatal day 60 (P60) to P90] were bred at our institute, as follows: Gad2-T2a-NLS-mCherry (catalog #023140), VGluT2-IRES-Cre (catalog #016963), Ai14(RCL-tdT)-D (catalog #007908); from The Jackson Laboratory; Drd1a-Cre, Drd2-Cre BAC from N. Heintz and P. Greengard (Rockefeller University, New York, NY); C. Gerfen (National Institute of Mental Health); and National Institute of Neurological Disorders and Stroke/GENSAT (www.gensat.org).

**Figure 1.**
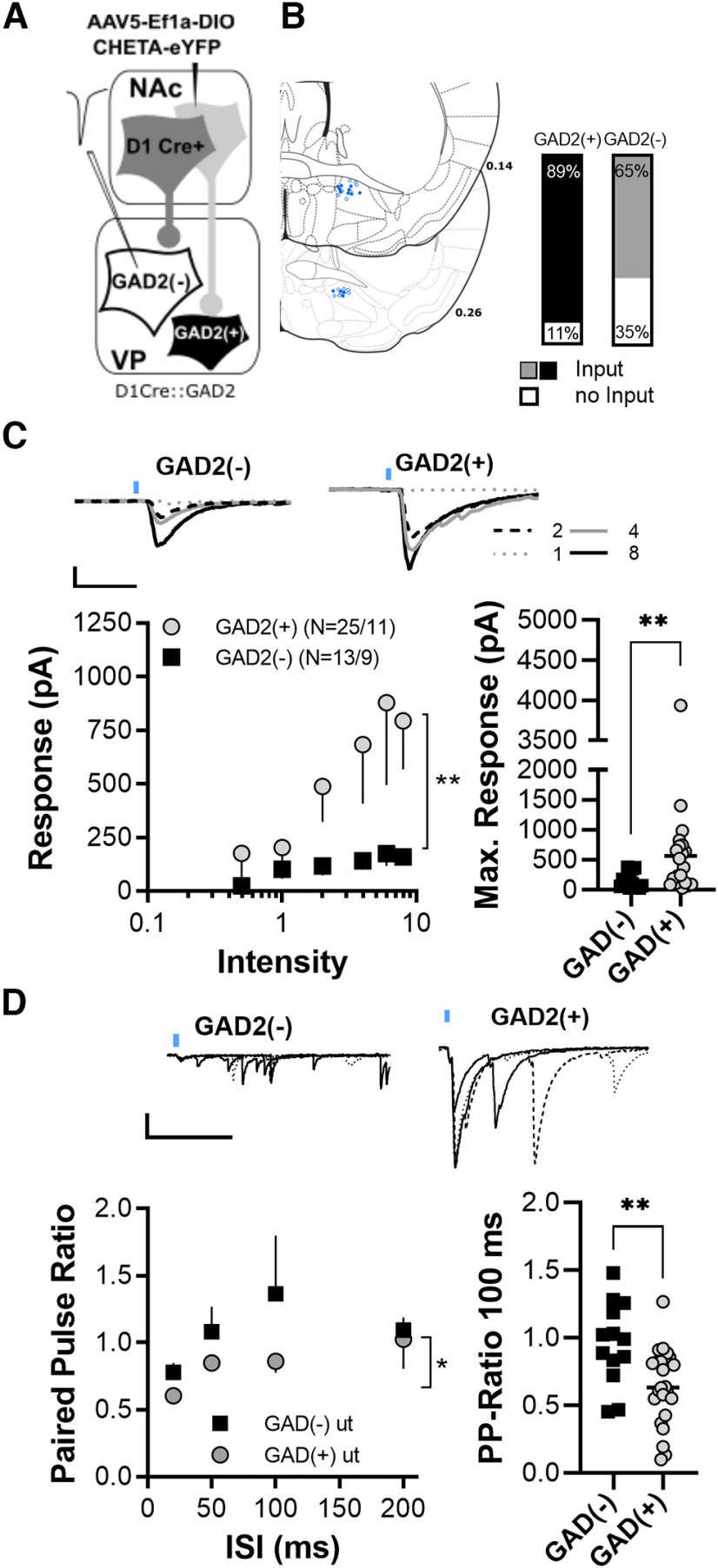
D1-MSN GABA_A_R-mediated transmission on GAD2(+) and GAD2(–) cells. ***A***, Illustration of the genetic circuit dissection. AAV5-EF1a-DIO-ChETA-EYFP was injected into the core of D1-Cre::GAD2 mice, which allowed selective optogenetic activation of D1-MSN terminals and recording of two distinct cell types [GAD2(+) and GAD2(–)] in the VP. ***B***, Left, Spatial distribution of recorded GAD2(+) cells (open symbols) and GAD2(–) cells (closed symbols) within D1-Cre::GAD2 mice. Connectivity plot on the left shows a significant higher percentage of GAD2(+) cells received D1-MSNs input. D1, 25 cells from 7 mice; D2, 23 cells from 9 mice. ***C***, Inset, Representative current traces of two GAD2(+) and GAD2(–) cells in response to increasing D1-D2-MSN stimulation. left: Input–output curves showing the relationship between laser stimulation intensity (in mW) and postsynaptic response amplitude. The response of GAD2(+) to stimulation of D1-MSN terminals was significantly stronger than the response of GAD2(–) cells. GAD2(+), 13 cells from 7 mice; GAD2(–), 12 cells from 9 mice. Right, Scatter plot showing the maximum response of GAD2(+) and GAD2(–) to D1-MSNs afferent stimulation. ***D***, Inset, Representative traces of GAD2(+) and GAD2(–) cells in response to ISI series. Left, Median paired-pulse ratio of evoked oIPSCs at different ISIs show higher paired-pulse ratios of GAD2(–) cells in response to D1-MSN terminal stimulation. GAD2(+), 11 cells from 6 mice; GAD2(–), 10 cells from 6 mice. Right, Scatter plot illustrating that paired-pulse ratios of GAD2(–) in response to a 100 ms ISI was significantly higher than GAD2(–) response. *p < 0.05; **p < 0.01. For representative viral expression and spread in the nucleus accumbens, see Extended Data Figure 1-1 (Extended Data Table 1-1, normality tests for all datasets).

**Figure 2.**
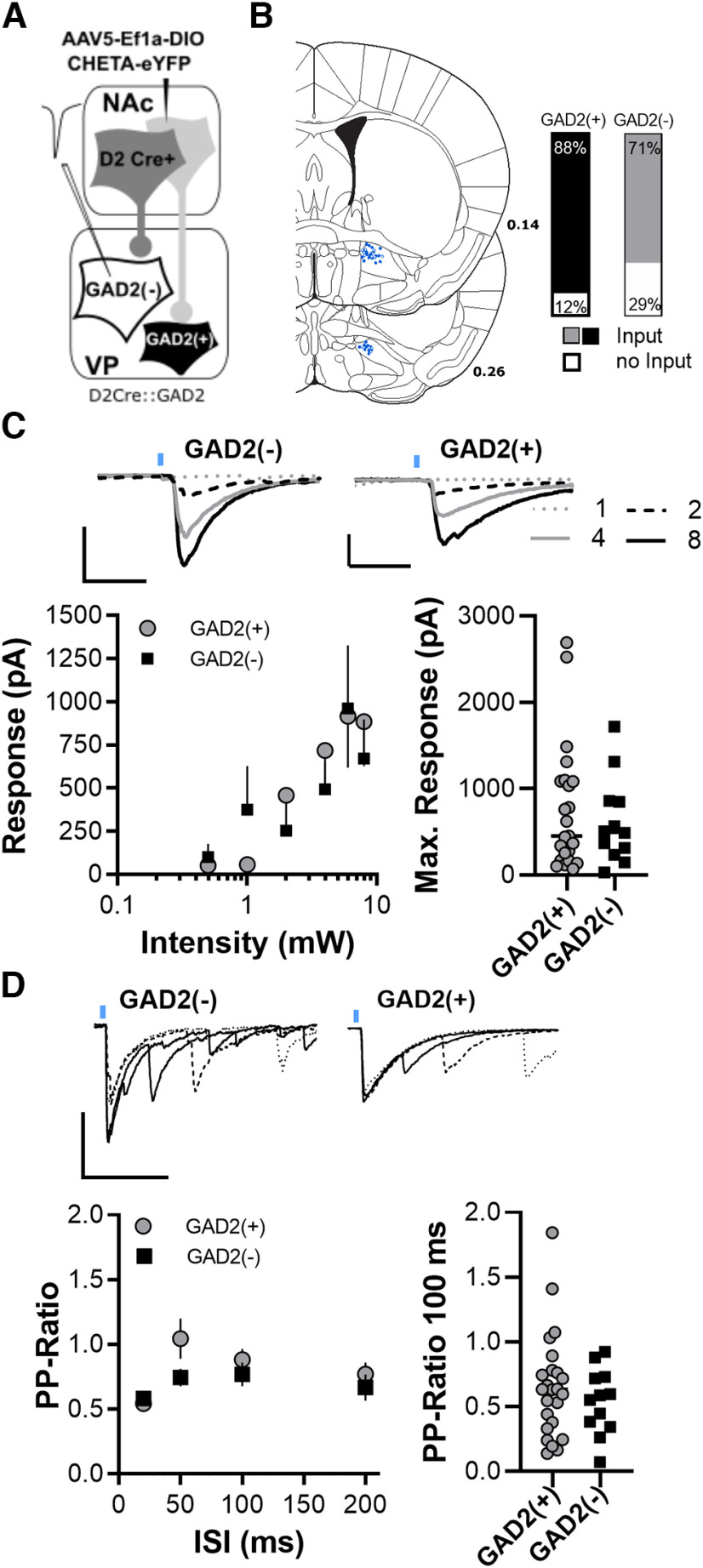
D2-MSN GABA_A_R-mediated transmission on GAD2(+) and GAD2(–) cells. ***A***, Illustration of the genetic circuit dissection. AAV5-EF1a-DIO-ChETA-EYFP was injected into the core of D2-Cre::GAD2 mice, which allowed selective optogenetic activation D2-MSN terminals in the VP. Responses of GAD2(+) cells (open symbols) and GAD2(–) cells (closed symbols) recorded under the anterior commissure. ***B***, Left, Spatial distribution of recorded GAD2(+) cells (open symbols) and GAD2(–) cells (closed symbols) within D2-Cre::GAD2 mice. Connectivity plot on the left shows that a significantly higher percentage of GAD2(+) cells received D2-MSNs input. GAD2(+), 27 cells from 7 mice; GAD2(–), 22 cells from 5 mice. ***C***, Inset, Representative current traces of a GAD2(+) and a GAD2(–) cell in response to increasing D2-MSN stimulation. Left, Input–output curves showing the relationship between laser stimulation intensity (in mW) and postsynaptic response amplitude. The response to stimulation of D2-MSN terminals was similar. GAD2(+), 25 cells from 11 mice; GAD2(–), 24 cells from 8 mice. Right, Scatter plot showing similar maximum response of the two recorded cell populations. ***D***, Inset, Representative traces of GAD2(+) and GAD2(–) cells in response to ISI series. Left, Median paired-pulse ratio of evoked oIPSCs at different ISIs showed higher paired-pulse ratios in response to D2-MSN terminal stimulation. GAD2(+), 15 cells from 8 mice; GAD2(–), 20 cells from 8 mice. Right, Scatter plot illustrating that paired-pulse ratios in response to 100 ms ISIs were significantly higher in response to D2-MSN terminal stimulations. *p < 0.05; **p < 0.01.

**Figure 3.**
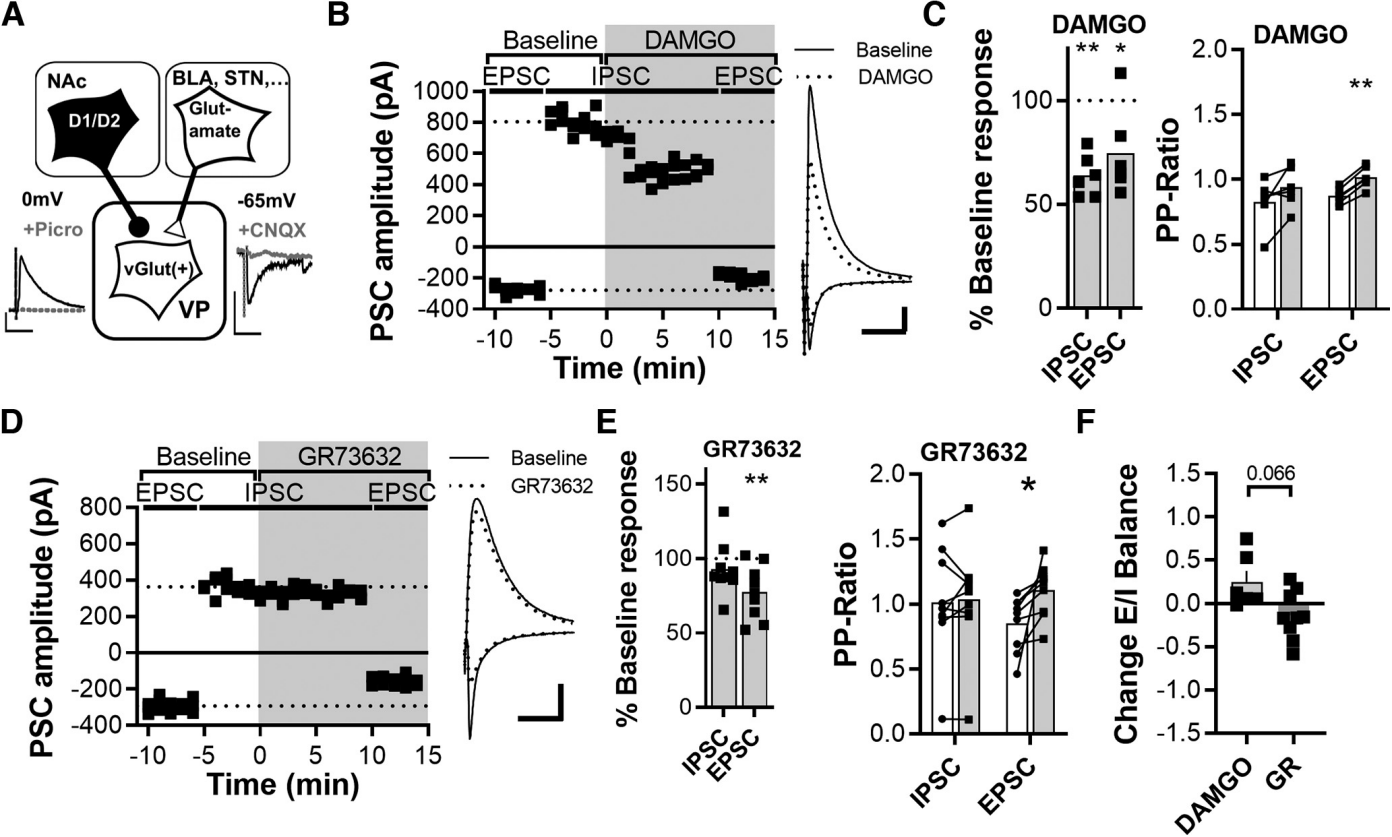
MOR and NK1R activation differentially modulate glutamate and GABA_A_R-mediated transmission onto VPGlutamate cells. ***A***, Schematic of the experimental protocol. VGluT2-IRES-Cre mice were crossed with Ai14 mice. Ai14 mice express robust tdTomato fluorescence in VPGlutamate cells following Cre-mediated recombination. VPGlutamate cells were recorded, and synaptic afferents were stimulated electrically. EPSCs and IPSCs were isolated biophysically by clamping the cells at –65 and 0 mV, respectively. eEPSCs and eIPSCs are were regularly blocked by DNQX and PTX, respectively (see example traces). For control experiments without agonists, see Extended Data Figure 3-1. ***B***, Example of serial recordings of EPSCs and IPSCs in a representative VPGlutamate cell. Both, EPSCs measured at −65 mV and IPSCs at 0 mV were decreased by the application MOR agonist DAMGO. ***C***, DAMGO inhibited elicited EPSCs and IPSCs (left) and significantly increased the paired-pulse ratio of EPSCs (right; pairwise comparison between preapplication and postapplication; 6 cells, 5 animals). ***D***, Example of serial recordings of EPSCs and IPSCs in a representative VPGlutamate cell. EPSCs measured at −65 mV but not IPSCs at 0 mV were decreased by application of NK1R agonist GR73632. ***E***, GR73632 application selectively inhibited elicited EPSCs but not IPSCs (left) and selectively increased the paired-pulse ratio of elicited EPSC (right; 9 cells, 5 animals). ***F***, Change in E/I ratio after DAMGO or GR73632 indicates a divergent modulation of inputs onto VPGlutamate cells. *p < 0.05; **p < 0.01.

**Figure 4.**
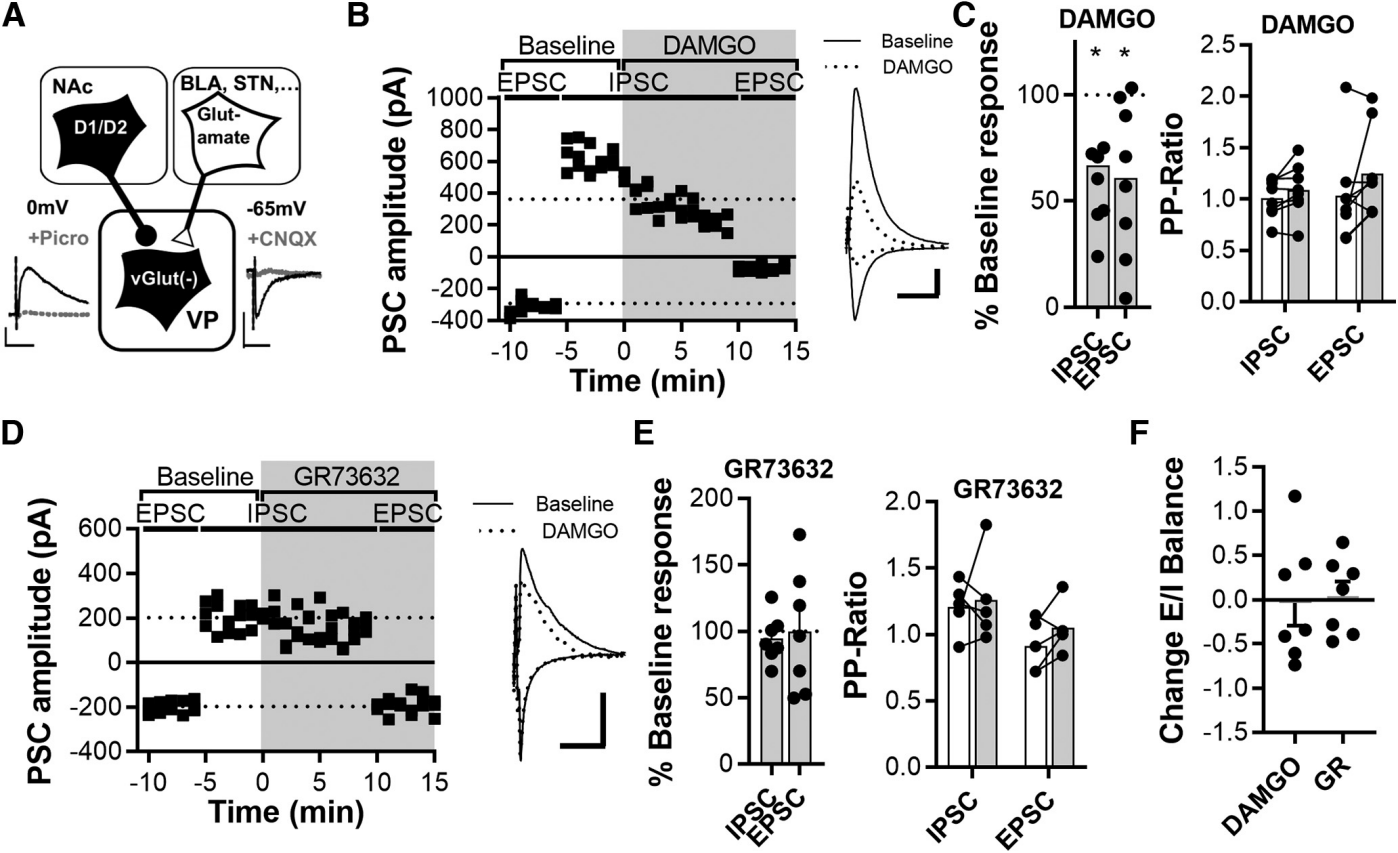
MOR activation but not NK1R activation inhibits both GABA_A_R and glutamate transmission onto VPGABA cells. ***A***, Schematic of the experimental protocol. VGluT2-IRES-Cre mice were crossed with Ai14 mice for robust tdTomato fluorescence in VPGlut(–) cells. VPGlut(–) cells (presumably VPGABA) were recorded, and synaptic afferents were stimulated electrically. EPSCs and IPSC were isolated biophysically by clamping the cells at –65 and 0 mV, respectively. EPSCs and IPSCs were regularly blocked by DNQX and PTX, respectively (see example traces). ***B***, Representative example of serial recordings of EPSCs and IPSCs in a representative VPGlut(–) cell. Both EPSCs measured at −65 mV and IPSCs measured at 0 mV were decreased by DAMGO. ***C***, DAMGO application inhibited both EPSCs and IPSCs (left) without significantly changing the paired-pulse ratio (right; 8 cells 6 animals). ***D***, Representative example of serial recordings of EPSCs and IPSCs in a representative VPGlut(–) cell. Neither EPSCs measured at −65 mV nor IPSCs measured at 0 mV were modulated by the NK1R agonist GR73632. ***E***, GR73632 application did not affect IPSC or EPSC amplitude (left) or paired-pulse ratios before and after drug application (right; 7 cells 4 animals). ***F***, No significant change in E/I ratio after DAMGO.

**Figure 5.**
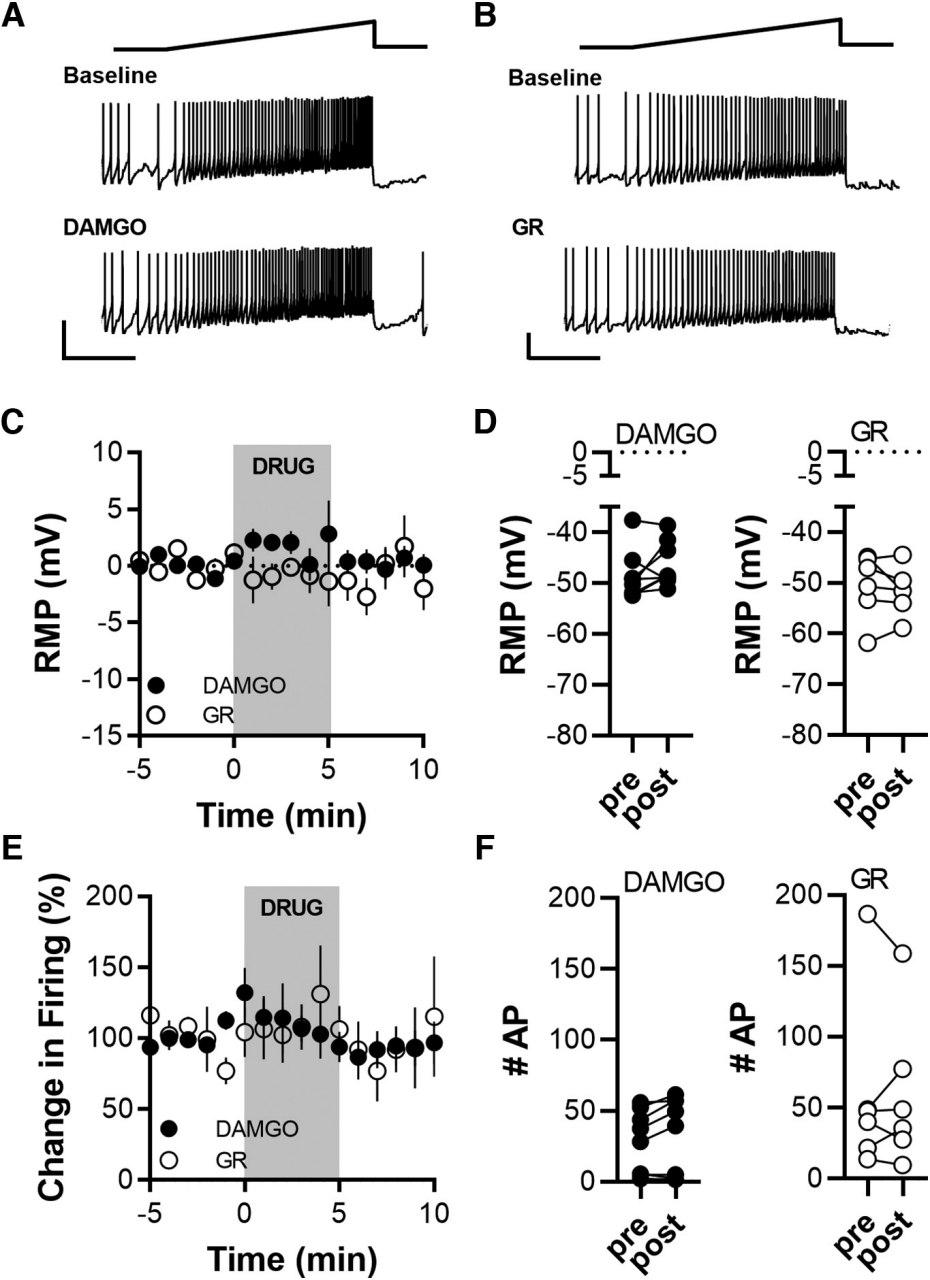
MOR and NK1R agonists do not modulate the excitability of VGluT2(+) cells. ***A***, Representative voltage traces of a VGluT2(+) cell in response to a 50 pA current ramp before and after DAMGO application. ***B***, Representative voltage traces of a firing VGluT2(+) cell before and after GR73632 application. ***C***, Time course of change in RMP before, during, and after drug application. ***D***, Pre-post paired comparisons of RMPs illustrate that neither MOR (DAMGO) nor NK1 (GR73632) activation significantly modulated the RMP of VGluT2(+) cells. DAMGO, 8 cells from 5 mice; GR73632, 6 cells from 4 mice. ***E***, Time course of change in firing rate during ramp-like depolarization before, during, and after drug application. ***F***, Pre-post paired comparisons illustrate that neither MOR (DAMGO) nor NK1 (GR73632) activation significantly modulated the elicited firing of VGluT2(+) cells. For input resistance before and after agonist application, see Extended Data Figure 5-1.

**Figure 6.**
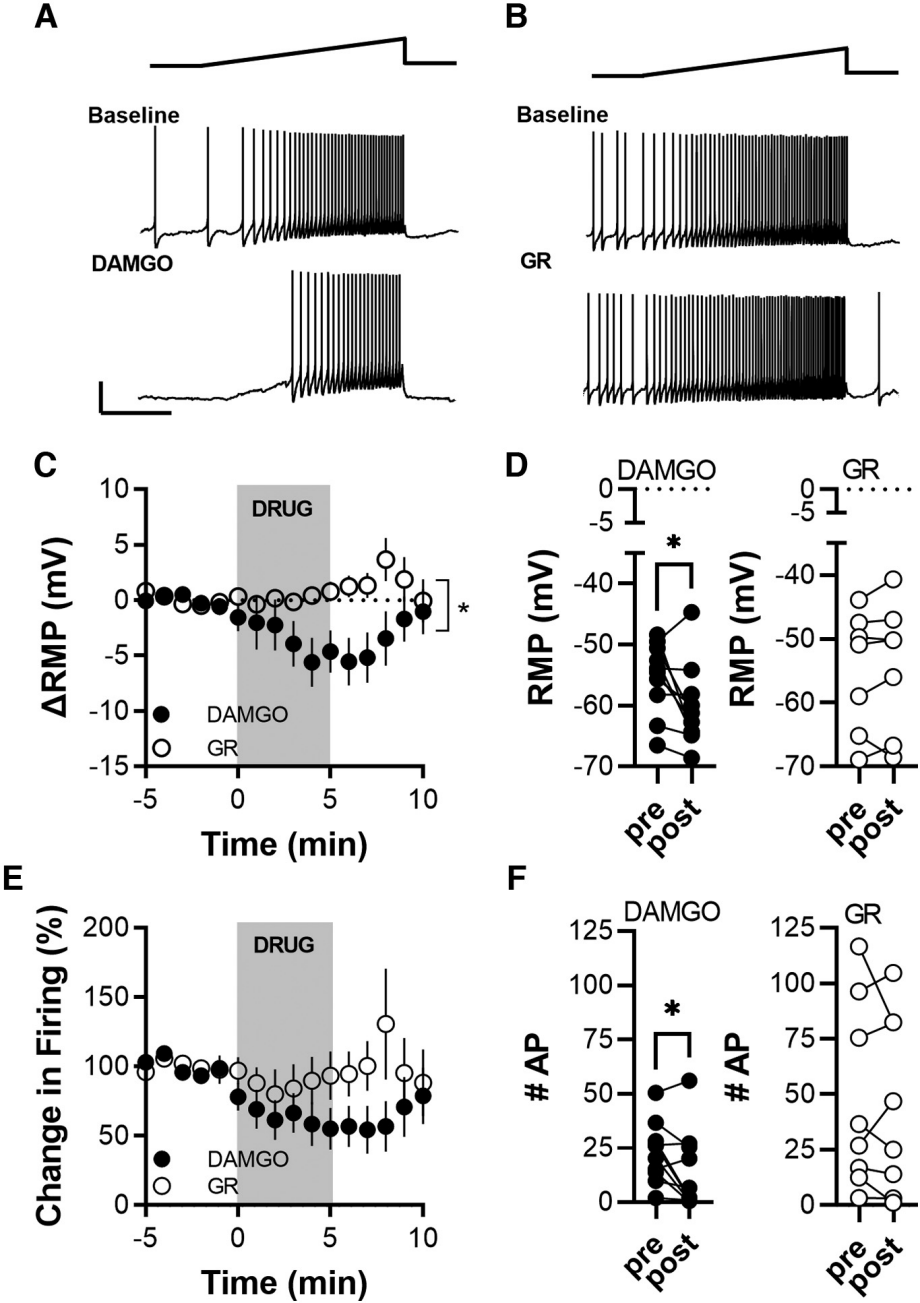
MOR but not NK1R activation hyperpolarizes VPGABA cells. ***A***, Representative voltage traces of a VPGlut(–) cell in response to a 50 pA current ramp before and after the DAMGO application illustrates the inhibition of firing. ***B***, Representative voltage traces of a firing VPGlut(–) cell before and after GR73632 application show no change. ***C***, Time course of change in RMP before, during, and after drug application. ***D***, DAMGO but not GR73632 significantly hyperpolarized the RMP of VPGlut(–) cells. DAMGO, 9 cells from 5 mice; GR73632, 10 cells from 6 mice. ***E***, Time course of change in firing rate before during ramp-like depolarization, before and after drug application. ***F***, DAMGO but not GR73632 significantly decreased the elicited firing of VPGlut(–) cells. *p < 0.05.

To investigate the afferent-specific GABA_A_R-mediated transmission onto VPGABA and VPGlutamate cells (Figs. 1, 2), Drd1a-Cre, Drd2-Cre mice that were cross-bred with Gad2-T2a-NLS-mCherry. To investigate cell type-specific neuropeptide modulation (Figs. 3-6) VGluT2-IRES-Cre mice were crossed with Ai14 mice.

10.1523/ENEURO.0404-22.2023.f1-1 Figure 1-1 Representative image of viral expression in the nucleus accumbens core of a GAD2xD1-cre mouse killed 4 weeks after injection of 280 nl of AAV5-Ef1a-DIO ChETA-EYFP. Aca, Anterior commissure; lV, lateral ventricle; acbC, nucleus accumbens core; acbSh, nucleus accumbens shell. Download Figure 1-1, DOCX file.

10.1523/ENEURO.0404-22.2023.tab1-1 Table 1-1 Kolmogorov–Smirnov normality tests. Download Table 1-1, DOCX file.

10.1523/ENEURO.0404-22.2023.f3-1 Figure 3-1 ***A***, ***B***, Depolarization to 0 mV and repolarization to 65mV did not significantly change the amplitude (***A***) or paired-pulse ratio (***B***) of EPSCs of VGluT(+) (VPGlutamate cells) or VGluT(–) (VPGABA cells). Download Figure 3-1, DOCX file.

Animals were group housed, fed *ad libitum*, and maintained in a humidity-controlled and temperature-controlled environment with 12 h light/dark cycle. Experiments were performed during the dark cycle. All experiments were conducted by the National Institute of Health *Guidelines for the Care and Use of Laboratory Animals*, and all procedures were approved by the Institutional Animal Care and Use Committee at our institute.

### Stereotaxic surgery

For viral microinfusion, Drd1a-Cre and Drd2-Cre mice::Gad2-T2a-NLS-mCherry mice (weight range, 20–30 g) were anesthetized with isoflurane (induction, 3–5% v/v; maintenance, 1–2% v/v). Analgesic (ketorolac, 2 mg/kg) was administered subcutaneously before surgery. Floxed AAV5-Ef1a-DIO ChETA-EYFP (7 × 10^12^ viral genomes/ml; 280 nl; Addgene) was delivered to the nucleus accumbens (anteroposterior, +1.5 mm; mediolateral, ±1.2 mm; dorsoventral, −4.4 mm) at a rate of 50 nl/min using pulled glass pipettes and a nanoinjector I (Drummond). Afterward, injectors were left in place for 10 min to allow for diffusion of the virus and then slowly retracted. Animals were given at least 4–5 weeks of recovery to allow for viral expression before slices were taken. A control virus lacking the hCHR2 transgene has been shown previously to be ineffective in stimulating transmitter release (data not shown; Extended Data Fig. 1-1, representative viral expression in the nucleus accumbens core).

### Whole-cell electrophysiology

Mice were anesthetized with a mixture of 100 mg ketamine and 10 mg xylazine/kg body weight (i.p.). Fresh VP slices (190 μm; model VT1200S vibratome, Leica) were cut in ice-cold (<4°C) cutting solution (92 *N*-methyl-d-glucamine, 20 HEPES, 25 glucose, 30 NaHCO_3_, 10 MgCl_2_, 5.0 ascorbic acid, 3.0 sodium pyruvate, 2.5 KCl, 1.2 NaH_2_PO_4_, and 0.5 CaCl_2_, pH 7.4; Parrilla-Carrero et al., 2021). Next, coronal slices were collected into a vial containing artificial CSF (aCSF; in mm: 126 NaCl, 1.4 NaH_2_PO_4_, 25 NaHCO3, 11 glucose, 1.2 MgCl2, 2.4 CaCl2, 2.5 KCl, 2.0 sodium pyruvate, and 0.4 ascorbic acid, bubbled with 95% O_2_ and 5% CO_2_) and 50 μm d-APV. Slices were kept at 22–24°C until they were used for recordings. During recordings, slices were constantly perfused with oxygenated aCSF and heated to 32°C (catalog #TC-344B, Warner Instruments). Neurons were visualized with a Zeiss Axioscope 2 FS plus microscope at 40×. A Multiclamp 700B amplifier (Molecular Devices) was used for all patch-clamp recordings. Membrane capacitance and input resistance were calculated automatically from a −2 mV pulse; Axograph X). Glass microelectrodes (tip resistance, 1.5–2.5 MΩ) were prepared using a PC-10 vertical puller (Narishige) and filled with internal solution according to distinct experiments (see below). Data were acquired at 10 kHz and filtered at 2 kHz using AxoGraph X software (AxoGraph Scientific). Recordings with unstable series resistance or with a series resistance >20 MΩ were discarded. Raw data were analyzed using AxoGraph X software.

#### Experiments to quantify GABA_A_R-mediated transmission from D1-MSN and D2-MSN terminals

Recordings of synaptic currents were performed in voltage clamp using a high-chloride internal solution to record GABA_A_R currents (in mm: 110 CsCl, 30 potassium gluconate, 10 HEPES potassium, 1 EGTA, 3 QX 314-Cl, 2.0 MgATP and 0.2 NaGTP, pH 7.2–7.3, 290 mOsm; Figs. 1, 2). Both, GAD2(+) (VPGABA, identified by their fluorescent soma) and GAD2(–) cells (presumed VPGlutamate, identified by the lack of fluorescence) were identified at 40× under a microscope. Recordings of synaptic transmission were started 10 min after invading the cell to allow for internal solution diffusion to remote dendrites. Excitatory synaptic transmission was blocked with 6-cyano-7-nitroquinoxaline-2,3-dione (10 μm) in the aCSF. IPSCs were evoked optically. The light pulse was produced by a 460 nm LED (Mightex) that was transmitted on the slice through the microscope objective. For input–output recordings, stimulation consisted of two pulses separated by 100 ms every 20 s. Stimulus intensity was increased from (3 ms, 0.5–8 mW) and at least six repetitions for each intensity recorded. For extended paired-pulse ratio, varying interstimulus intervals (25, 50, 100, and 200 ms) were tested every 20 s with six repetitions each. The stimulation intensity was set to evoke an IPSC with amplitudes within the dynamic range of the cells (i.e., 30–70% of maximal IPSCs; 200–900 pA).

#### Experiments to quantify the modulation of transmitter release by neuropeptides

Recordings of synaptic currents were performed in voltage-clamp configuration using a cesium-methanesulfonate-based internal solution (in mm: 115 Cs+methanesulfonate, 10 HEPES, 1 EGTA, 1 MgCl2, 1 NaCl, 2 Mg2+-ATP, 0.3 Na^+^-GTP, 3 QX 314-Cl, and 10 BAPTA-tetracesium, with 290 mOsm, at pH 7.4; Figs. 3, 4). We used a high concentration of BAPTA in the internal solution to chelate calcium and to prevent calcium signaling and potential synaptic plasticity induced by depolarization (Francis et al., 2019). Afferents were stimulated electrically via a bipolar stimulation electrode positioned 200–300 µm dorsomedial to the recorded cell. The stimulation intensity was set to evoke an EPSC with amplitudes within the dynamic range of the cells (i.e., 30–70% of maximal IPSCs; 200–900 pA). Biophysically isolated basal AMPAR evoked EPSCs (eEPSCs) at –65 mV and basal GABA_A_ evoked IPSCs (eIPSCs) at 0 mV were recorded for a stable baseline. We then applied DAMGO (1 μm) or GR73632, (0.2 µm). After 10 min of drug application, we subsequently recorded AMPAR eEPSCs at −65 mV. eEPSCs and eIPSCs are regularly blocked by DNQX and picrotoxin (PTX), respectively.

#### Neuropeptide modulation of excitability

Recordings of resting membrane potential (RMP) and action potentials were performed in current-clamp configuration using a potassium-gluconate-based internal solution (in mm: 135 K^+^ gluconate, 5 KCl, 1 MgCl_2_, 1 EGTA, 0.3 CaCl_2_, 10 HEPES, 2 Na^2+^ ATP, and 0.3 Na^+^ GTP, at pH 7.2–7.3 and 290 mOsm).

Statistical analyses of data were performed with GraphPad Prism (GraphPad Software) using tests indicated in the main text. Nonparametric tests were used if data distributions for comparisons of groups did not pass the Kolmogorov–Smirnov normality test (Extended Data Table 1-1).

## Results

### GABA_A_R-mediated transmission from D1-MSNs on GAD2(+) and GAD2(–) cells

First, we aimed to probe the synaptic strength between NAc D1-MSNs on VPGABA and VPGlutamate cells. We microinjected a cre-dependent virus carrying a ChETA variant of channelrhodopsin into the NAc core of Drd1a-Cre::Gad2-T2a-NLS-mCherry (Fig. 1*A*). Adult mice were killed 4 weeks after virus injection and either GAD2(+) cells (presumed VPGABA cells) or GAD2(–) (presumed VPGlutamate cells) were recorded within the terminal field of transfected MSNs within the VP (Fig. 1*B*). Optical stimulation of D1-MSN terminals was used to evoke GABAergic inhibitory postsynaptic responses [optically evoked IPSCs (oIPSCs)]. We found that a higher percentage of recorded GAD2(+) cells than GAD2(–) cells received input from D1-MSN afferents (Fig. 1*B*; χ^2^ = 16.26, *p* < 0.01). Figure 1*C*, left, shows the oIPSCs of responsive GAD2(+) and GAD2(–) cells in response to increasing laser intensity. The strength of D1-MSN input on GAD2(+) cells was significantly stronger than that for D1-MSN input on GAD2(–) cells (Fig. 1*C*, left; *F*_(1,132)_ = 10.33, *p* < 0.01; cell type, two-way ANOVA) and the maximum oIPSC amplitudes were higher for GAD2(+) cells (Fig. 1*C*, right; *p* < 0.01, Mann–Whitney test). To estimate whether the weaker synaptic strength of D1-MSNs onto GAD2(–) could be explained by lower GABA release probability, we quantified paired-pulse ratios in response to four different interstimulus intervals (ISIs) and found a strong trend toward higher ISIs for D1-MSN to GAD2(–) synapses (Fig. 1*D*, left; *F*_(1,81)_ = 3.486, *p* = 0.065, cell type, two-way ANOVA). An additional set of cells from a previous experiment that was only tested for a fixed ISI of 100 ms also showed significantly higher paired-pulse ratios for D1-VPGlutamate responses (Fig. 1*D*, right; *t*_(35)_ = 3.187; *p* < 0.01, unpaired *t* test). These results indicate that the weaker synaptic strength of D1-VPGlutamate is likely caused by a lower GABA release probability from these synapses.

### GABA_A_R-mediated transmission from D2-MSN on GAD2(+) and GAD2(–) cells

Similar to the previous section, we next probed the functional connectivity between NAc D2-MSNs on VPGABA and VPGlutamate cells via optogenetics in Drd2a-Cre::Gad2-mCherry mice (Fig. 2*A*). GABAergic oIPSCs were recorded from GAD2(+) and GAD2(–) cells in the fluorescent terminal fields of VP slices (Fig. 2*B*). We found that a higher percentage of VPGABA responded to D2-MSN terminal stimulation (Fig. 2*B*; χ^2^ = 8.866, *p* < 0.001). Input–output curves testing the relationship between laser power and oIPSC showed no significant difference in the strength of GABAergic transmission of D2-MSN inputs onto GAD2(+) and GAD2(–) (Fig. 2*C*, left; *F*_(1,161)_ = 0.0539, *p* = 0.871; cell type, two-way ANOVA). The maximum oIPSC amplitudes were also similar for responses to stimulation of D1-terminals and D2-terminals (Fig. 2*C*, right; *p* > 0.05, Mann–Whitney test). The paired-pulse ratios were similar as well, indicating no difference in release probability between D2-VPGABA and D2-VPGlutamate synapses (Fig. 2*D*, left; *F*_(1,116)_ = 2.568, *p* = 0.118; afferent type, two-way ANOVA; Fig. 2*D*, right; *p* = 0.567, Mann–Whitney test).

### Effect of stimulating NK1 and μ-opioid receptors on VGluT2(+) cells

In addition to GABA, D1-MSNs corelease the neuropeptides substance P and dynorphin (activating NK1Rs and κ opioid receptors, respectively), and D2-MSNs corelease enkephalin and neurotensin (activating μ/δ-opioid receptors and neurotensin receptors, respectively). The experimental procedure to determine whether the pharmacological activation of these receptors can differentially regulate inhibitory and excitatory neurotransmission onto VGluT2(+) is illustrated in Figure 3*A*. Fluorescent cells in VGluT2::Ai14 reporter mice were recorded, and afferents were stimulated electrically. AMPAR-mediated currents were biophysically isolated by clamping cells at the reversal potential for GABA_A_Rs (−65 mV), and GABA_A_R-mediated transmission was isolated by clamping cells at the reversal potential for AMPAR and NMDAR (0 mV). eEPSCs and eIPSCs were regularly blocked by DNQX and PTX, respectively (see example traces). The time course and response of one representative experiment with the MOR agonist DAMGO is illustrated in Figure 3*B*. The VGluT2(+) neuron was clamped at −65 mV to record EPSCs, and, after a stable baseline of 5 min, cells were slowly depolarized to 0 mV to record IPSCs. DAMGO was applied after a baseline recording of 5 min. After 10 additional minutes of recording at 0 mV, cells were clamped at –65 mV for at least 5 more minutes. With a set of control experiments, which followed the time course of depolarization and repolarization, we verified that the EPSCs amplitude of VPGlutamate cells was not significantly changed by prolonged depolarization (Extended Data Fig. 3-1*A*). Figure 3*C* shows that DAMGO application significantly depressed evoked IPSCs and EPSCs onto VGluT2(+) cells (Fig. 3*C*, left; *t*_(5)_ = 8.801, *p* < 0.01; *t*_(5)_ = 2.963, *p* < 0.05; Student’s *t* test). The paired-pulse ratio of responses was evaluated with two paired pulses separated by a 100 ms interstimulus interval. Evoked IPSCs showed a trend toward increase in paired-pulse ratio after DAMGO application, and evoked EPSCs showed a significant increase (Fig. 3*C*, right; *t*_(5)_ = 2.023, *p* = 0.1; *t*_(5)_ = 13.06, *p* < 0.01; paired *t* test), indicating that the inhibition of EPSCs was based on the decrease of glutamate release probability. Figure 3*D* shows the time course and the response of one representative experiment with the NK1R agonist GR73632, which was applied after a baseline recording of 5 min. IPSCs were not affected by GR73632 application while EPSCs were significantly decreased (Fig. 3*E*, left; *t*_(8)_ = 1.188, *p* = 0.269; *t*_(8)_ = 3.758, *p* < 0.01; Student’s *t* test). The significant increase in paired-pulse ratio indicates a decrease glutamate release probability after GR73632 application (Fig. 3*E*, right; *t*_(5)_ = 0.418, *p* = 0.687; *t*_(8)_ = 2.753 *p* < 0.05; paired *t* test).

Next, we calculated the ratio of excitation to inhibition (E/I ratio) by dividing the average amplitude of EPSC (at −65 mV) by the average amplitude of IPSC (at 0 mV). Figure 3*F* illustrates the change in E/I ratio after neuropeptide application. Overall, GR73632 showed a strong trend toward a significant decrease of the E/I ratio compared with the DAMGO application (Fig. 3*F*; *p* = 0.06, Mann–Whitney test). These results imply that substance P released from D1 terminals could shift the balance from neurotransmission toward inhibition.

### Effect of stimulating NK1Rs and μ-opioid receptors on VGluT2(–) cells

To determine whether DAMGO and GR73632 are differentially regulating inhibitory and excitatory neurotransmission onto VGluT2(–) cells, we recorded nonfluorescent cells in the VP of VGluT2::Ai14 reporter mice (Fig. 4*A*). Figure 4*B* shows a representative example of a VGluT2(–) cell clamped at –65 mV to record voltage-isolated EPSCs. After obtaining a stable baseline, cells were slowly depolarized to 0 mV to record IPSCs, and after 5 min of baseline, the MOR agonist DAMGO was applied. Ten minutes later, the cell was clamped at –65 mV for at least 5 more minutes. With a set of control experiments, which followed the time course of depolarization and repolarization, we verified that the EPSC amplitude of VPGABA cells was not significantly changed by prolonged depolarization (Extended Data Fig. 3-1). DAMGO application significantly depressed elicited IPSCs and EPSCs onto VPGABA cells (Fig. 4*C*, left; *t*_(7)_ = 2.668, *p* < 0.05; *t*_(7)_ = 3.029, *p* < 0.05; Student’s *t* test). Evoked IPSCs and EPSC showed no change in paired-pulse ratio after DAMGO (Fig. 4*C*; IPSC, *p* = 0.11; EPSC, *p* = 0.11; Wilcoxon test). Figure 4*D* shows a representative recording of a VGluT2(–) cell before and during GR73632 application. Application of GR73632 did not affect response strength (Fig. 4*E*, left; *t*_(6)_ = 0.823, *p* = 0.442; *t*_(6)_ = 0.0111, *p* = 0.992; Student’s *t* test) or paired-pulse ratio (Fig. 4*E*; IPSC, *p* > 0.99; EPSC, *p* = 0.18; Wilcoxon test) of VGluT2(–) cells.

GR73632 and DAMGO applications did not shift the E/I ratio in VGluT2(–) cells (Fig. 4*F*; *p* = 0.802; Mann–Whitney test).

### Effect of stimulating NK1Rs and μ-opioid receptors on VGluT2(+) excitability

In previous experiments, we interrogated the effect of neuropeptides on GABA and glutamate transmission. Next, we wanted to quantify potential effects on VGluT2(+) such as RMP and firing rate. To this aim, cells were recorded at resting membrane potential in the current clamp and every 20 s were depolarized with a ramp pulse of current increasing from 0 to 50 pA over 5 s (Fig. 5*A*,*B*, top, depolarizing ramp, middle panels, one representative baseline response). After a 5 min baseline, DAMGO or GR73632 was applied, and the impact on RMP and firing rate in response to the ramp depolarization was quantified for a 5 min baseline and 2–6 min after drug application (Fig. 5*A*,*B*, bottom panels, a representative response 5 min after drug application). Neither drug changed RMP (Fig. 5*C*,*D*; DAMGO, *p* = 0.250; GR73632, *p* = 0.688; Wilcoxon test) or firing rate (Fig. 5*E*,*F*; DAMGO, *p* = 0.109; GR73632, *p* = 0.982; Wilcoxon test) of recorded VGluT2(+) cells.

**Figure 7.**
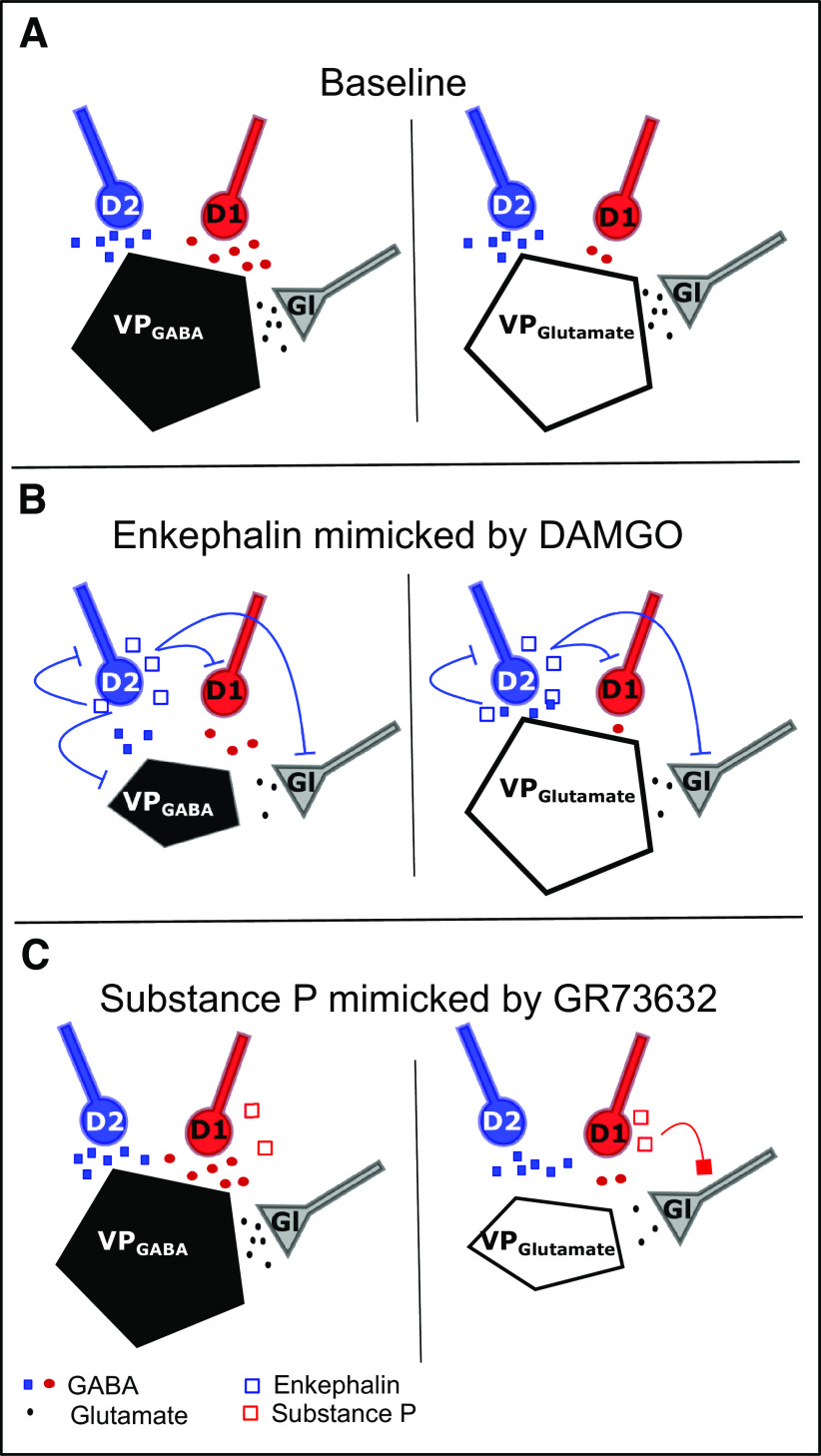
GABA and neuropeptides can fine-tune information flow in the VP in a cell-type, transmitter-specific manner. ***A***, Under baseline conditions, VPGABA cells receive D1-MSN and D2-MSN GABA_A_R input of similar strength. VPGlutamate cells receive significantly lower GABA_A_R-mediated transmission from D1-MSN terminals. ***B***, The activation of MOR inhibits both GABA and glutamate transmission onto VPGABA to a similar extent. Additionally, MOR activation hyperpolarizes VPGABA cells. MOR activation only inhibited neurotransmission onto VPGlutamate cells; it had no postsynaptic effect. ***C***, NK1R activation modulated neither the neurotransmission nor the excitability of VPGABA cells. NK1R activation inhibits EPSCs onto VPGlutamate cells.

10.1523/ENEURO.0404-22.2023.f5-1 Figure 5-1 ***A***, DAMGO but not GR73632 significantly reduced the input resistance (Rin) of VPGlut(–) cells. *p* = 0.044, paired *t* test. DAMGO, 5 cells from 4 mice; GR, 6 cells from 4 mice. ***B***, Neither DAMGO nor GR73632 affected the Rin of VPGlut(+) cells. DAMGO, 6 cells from 4 mice; GR, 2 cells from 2 mice. Note that sample size is different from experiments in Figures 6 and 7 because a membrane test using a –10 pA hyperpolarization step was not performed for all experiments. Download Figure 5-1, DOCX file.

### Effect of stimulating NK1Rs and μ-opioid receptors on VGluT2(–) excitability

Next, we quantified the effect of DAMGO and GR73632 on VGluT2(–) RMP and firing rate. Figure 6, *A* and *B*, shows representative traces of two experiments with DAMGO and GR73632 respectively. Application of DAMGO significantly hyperpolarized the RMP of VGluT2(–) cells compared with GR73632 application (Fig. 6*C*; main drug effect: *p* ≤ 0.001, *F*_(1,264)_ = 30.56; interaction drug × time: *p* = 0.04, *F*_(15,264)_ = 1.766; two-way ANOVA) and compared with baseline (Fig. 6*D*; *p* = 0.035, *t*_(9)_ = 2.453). The DAMGO-induced hyperpolarization was accompanied by a significant decrease in input resistance (Extended Data Fig. 5-1) and significantly decreased the firing rate (Fig. 6*E*; *p* = 0.043, *t*_(9)_ = 2.350), while the firing was not affected by the application of GR73632 (Fig. 6*F*; *p* = 0.02, *t*_(7)_ = 0.545). GR73632 also did not affect the input resistance of VGluT2(–) cells.

## Discussion

### Cell type-specific GABAAR signaling in the ventral pallidum

We found that the D1-MSN GABA release onto GABA_A_Rs of GAD2(–) cells was significantly weaker than on GAD2(+) cells. D2-MSN GABA_A_Rs mediated transmission was equally strong on GAD2(+) and GAD2(–) cells. The GABA release probability of D1-GAD2(–) synapses was significantly lower compared with D1-GAD2(+) synapses, indicating a presynaptic restriction of GABA release. These results are surprising because they demonstrate that, contrary to our expectations, D1-MSNs seem to exert higher inhibitory control over VPGABA cells than over VPGlutamate cells, at least at levels of low activity when neuropeptide release would be expected to be low (Francis et al., 2019; see below). Since the strength of synapses is not static but can be modulated by experience, the weak D1-GAD2(–) synapses may serve as a substrate for future synaptic potentiation. Indeed, we and others have recently shown that withdrawal from chronic cocaine exposure selectively depresses D2-MSN synapses in the ventral pallidum via presynaptic MOR-dependent mechanisms (Creed et al., 2016; Heinsbroek et al., 2017). Furthermore, Creed et al. (2016) found that cocaine selectively potentiates D1-MSN synapses in the VP and that rectification of this plasticity inhibits cocaine sensitization. These results indicate that while the inhibitory control of D1-MSNS over VPGlutamate cells might be significantly lower in physiological conditions, it might become more important in disease states such as addiction and depression.

Our findings that a similar percentage of GAD2(–) cells receive D1-MSN and D2-MSN input seemingly contradicts a recent rabies tracing study from our laboratory that showed more D1-MSNs than D2-MSNs innervating VPGlutamate cells (Heinsbroek et al., 2020). It cannot be excluded that some rabies tropism toward D1-MSN existed, overestimating D1-MSN input onto VPGlutamate cells. Furthermore, while rabies tracing allows quantification of direct synaptic connections between two cells, it does not evaluate the strength of these connections. Some of these D1-MSN synapses may be weak or silent and not detected by whole-cell patch-clamp recordings. It could also be that some of these synapses are purely neuropeptidergic (Cifuentes and Morales, 2021). A similar noncanonical segregation between glutamate and dopamine release has also been demonstrated in the VTA (Cifuentes and Morales, 2021).

### Cell type-specific neuropeptide signaling in the ventral pallidum

To examine how coreleased neuropeptides modulate neurotransmission and excitability of VPGABA and VPGlutamate, we focused on activating either MOR (via the MOR agonist DAMGO) or NK1R (via the NK1R agonist GR73632) because they are major targets of enkephalin and substance P in the VP (Kupchik et al., 2014; Vanderah and Sandweiss, 2015) and their action within the VP is implicated in motivated behaviors (Hasenöhrl et al., 2000; Smith and Berridge, 2005; Tang et al., 2005). We found that the administration of DAMGO inhibited GABAergic and glutamatergic afferents on both VPGABA and VPGlutamate to a similar extent. Interestingly, DAMGO hyperpolarized and inhibited action potential firing only in VPGABA cells. The DAMGO-induced hyperpolarization was accompanied by a significant decrease in input resistance, which indicates that the hyperpolarization is mediated by a canonical increase of potassium conductance (North et al., 1987). We furthermore found that GR73632 has the potential to downregulate the activity of VPGlutamate cells by selectively inhibiting glutamate transmission on this cell type (Fig. 7*C*,*D*). The significant increase in paired-pulse ratio indicates a presynaptic locus of the NK1R-mediated inhibition of glutamate transmission. NK1R have been reported to be Gs-protein coupled and mainly expressed on cholinergic neurons in the VP (Mengual et al., 2008). The observed presynaptic depression of glutamatergic terminals could be accomplished via a heterosynaptic process involving acetylcholine release and presynaptically expressed Gi-protein-coupled M2 receptors. Alternatively, presynaptic NK1R activation could trigger a nitric oxide synthase-dependent LTD, as described for the cerebral cortex (Matsumura et al., 2021).

Neuropeptides are stored in dense core vesicles, and neuropeptide release usually requires higher synaptic activity compared with GABA release (Francis et al., 2019). Hence the corelease of GABA and neuropeptides from the same synaptic terminals can multiplex the synaptic control over their postsynaptic target in an activity-dependent manner with GABAergic inhibition acting at low presynaptic activity and neuropeptidergic modulation acting at higher or sustained presynaptic activity. The potential of activity-dependent multiplexing was illustrated in an elegant study by Soares-Cunha et al. (2020), who found that D2-MSN stimulation elicits opponent behavioral responses, depending on the stimulation pattern. A brief optogenetic simulation of D2-MSNs inhibited VP neurons and conditioned place preference, whereas a prolonged stimulation elicited conditioned place aversion (CPA). CPA could be blocked by the administration of the δ opioid receptor antagonist naltrindole (Soares-Cunha et al., 2020).

### Limitations of this study and potential future directions

One caveat of this study is that for the afferent and cell type-specific experiments (Figs. 1, 2), we used an indirect approach using tomato^–^ cells GAD2(–) to identify VPGlutamate, which could result in not all GAD2(–) cells recorded being glutamatergic neurons. Cholinergic neurons constitute a small (<5%) group of neurons in the dorsolateral VP, where our recordings were made, and, similar to the striatum (Tepper and Bolam, 2004), cholinergic neurons in the VP can also be discriminated from other cell types because of their significantly larger soma size (Bengtson and Osborne, 2000). It is also possible that GABA cells expressing low levels of GAD2 or the genetic reporter could be mischaracterized as VPGlutamate. However, a recent RNA scope analysis by Stephenson-Jones et al. (2020) reveals negligible overlap between GAD2-expressing and VGluT2-expressing cells. Although we cannot completely rule out some mislabeling, similar to the common practice of identifying D1-MSNs or D2-MSNs based on a lack of reporter presence in mice expressing a reporter in one or the other subpopulation, it seems appropriate to assume that GAD2(–) cells are largely VPGlutamate.

Although there is only a small subpopulation of VP neurons that are not VPGlutamate and VPGABA cells, there are several distinct subpopulations of GABA neurons that can be subdivided based on molecular markers such as preproenkephalin or parvalbumin (Root et al., 2015; Knowland et al., 2017; Heinsbroek et al., 2020), or are based on their projection targets, and drive distinct affective and reward behaviors (Knowland et al., 2017; Vachez et al., 2021). While the investigation of these multiple subpopulations is beyond the scope of this article, the results from such studies will allow for a more complete understanding of how the accumbens–VP subcircuits modulate reward and aversion.

Another caveat of this study is that only synthetic analogs of the coreleased neuropeptides were used in relatively high doses. Accordingly, our experiments may not accurately reflect the *in vivo* release of enkephalin and substance P. Different affinities of MORs expressed on different synaptic terminals could allow for an even more nuanced tuning of the modulation of transmitter release. Emerging techniques to visualize *in vivo* neuropeptide release (Jullié et al., 2022) in combination with cell type-specific activity monitoring should be useful in more precisely understanding the effects of endogenous peptide release. While we only looked at the activation of MORs, enkephalin does also act on δ opioid receptors, which are exclusively expressed on D2-MSN synapses in the ventral pallidum (Creed et al., 2016; Soares-Cunha et al., 2020) and likely also control information flow through the VP.

Our study focused on fast ionotropic GABA transmission via GABA_A_ receptors, but metabotropic GABA_B_ receptors are also present in the VP. However, GABA_B_ expression is low and constrained to cell bodies of non-GABAergic neurons (Margeta-Mitrovic et al., 1999). Nonetheless, our study cannot exclude that GABA_B_ receptors may play a cell type-specific role in modulating neurotransmission and excitability.

Finally, it should be noted that the action of different neuropeptides strongly depends on the VP subcompartment being studied. For example, MOR activation via DAMGO in the caudal VP elicits appetitive responses, whereas MOR activation in the more rostral regions elicits aversive responses (Smith and Berridge, 2005). Also, VP inactivation of the rostral VP inhibits cue-induced reinstatement, whereas inhibition of the caudal VP had no effect (Mahler et al., 2014). Because our recordings strictly focused on the subcommisural region (bregma between 0.3 and 0.14 mm), it will be important in future studies to investigate neuropeptidergic effects on neurotransmission and excitability along the rostrocaudal axis.

### Conclusions

Overall, our findings show that D1-MSN and D2-MSN afferents in the VP have the potential to exert bidirectional control over VPGABA and VPGlutamate neurons with enkephalin released from D2-MSNS potentially exerting stronger inhibition and VPGABA (via an additional postsynaptic inhibition that is lacking on VPGlutamate cells; Fig. 7). Our data indicate that the effect of substance P on both cell types could also be activity dependent where GABA transmission is stronger on VPGABA under low activity and glutamate transmission onto VPGlutamate can be inhibited via substance P release under high activity.

Understanding the nuanced subcircuit regulation between the accumbens and VP is critical for integrating the emerging understanding that the two distinct subpopulations within each nucleus (accumbens and VP) differentially control whether an animal will approach or avoid a stimulus. Further experimentation based on our initial studies (as outlined above), will ultimately parse subcircuits based on neurotransmitter expression and projection patterns; thereby, potentially providing a rationale for therapeutic regulation in neuropsychiatric disorders where symptoms include maladaptive reward or avoidance behaviors, such as substance use disorders or post-traumatic stress disorder, respectively.

## Synthesis

Reviewing Editor: Jean Christophe Poncer, INSERM Decisions are customarily a result of the Reviewing Editor and the peer reviewers coming together and discussing their recommendations until a consensus is reached. When revisions are invited, a fact-based synthesis statement explaining their decision and outlining what is needed to prepare a revision will be listed below. The following reviewer(s) agreed to reveal their identity: Frank Meye, Manuel Mameli.

Both reviewers and I found that your report on differential innervation of glutamatergic and GABAergic VP neurons by MSNs expressing D1R vsD2R is timely and important to the field. They found the experiments to be generally well designed and supportive of the main findings of the study. However, they raised a number of questions about the data and their interpretation that need to be addressed.

The reviewers and I believe that the following points are particularly important to address with additional experiments or analyses:

1. The manuscript suffers from a lack of histological data showing the specificity and spread of the viral infection.

2. It is unclear whether Cheta expression across slices was homogeneous and thus whether direct comparison of input/output relationships is valid. It is also questionable whether the comparison of input/output in slices from animals with different genetic backgrounds is valid. This last point should at least be discussed in the manuscript, or tested experimentally.

3. The experiments testing the effects of MOR and NK1R were performed under conditions where D1R and D2R inputs were not differentiated. We believe it would be important to explore possible differences between these two inputs.

4. In experiments shown in Fig. 3 and 4, we feel it is important to verify that changes in EPSC amplitude upon pharmacological manipulations are independent of the prolonged depolarization (as used for testing IPSC amplitude) or some time-dependent drift in synaptic transmission.

--

Reviewer #1

The current study uses path clamp electrophysiological recordings in mouse brain slices, together with optogenetics and pharmacology, to evaluate the connectivity between two separate populations of nucleus accumbens medium spiny neurons (D1R and D2R subtypes) with two subtypes of ventral pallidal neurons (GABA and glutamate). Aside from ionotropic GABAAR mediated transmission in this circuitry, the authors evaluate the effects that two relevant GPCRs (i.e. GPCRs for some of the peptides NAc neurons can corelease) may have in regulating VP cellular subtypes. The authors find that D1R NAc neurons preferentially synapse onto VP GABA over VP putative glutamate neurons. Instead D2R NAc neurons do not have such a preferential innervation pattern. Mu-opioid receptor activation reduces both GABA and glutamate input onto both VP neuron subtypes, and hyperpolarizes VP GABA cells. The effects of NK1R activation was more limited. Overall the study offers some nice further insight in NAc-VP connectivity through a substantial dataset. Some matters do however need to still be addressed:

1. There is generally no histology shown for the experiments. The injections are said to be aimed at the NAc core (at a given set of coordinates), but it is unclear whether they also in practice ended up being selective to the core. It is important to show representative images of this per experiment type (e.g. per different mouse strain injected), as also different mouse lines can have differences in brain anatomy to the point that the same coordinates would end up differently targeting NAc sub-territories across experiments.

2. The authors make comparisons across transgenic genotypes. For instance comparing connectivity of NAc D1 and D2 MSNs with VP neuronal subtypes in such features as maximal IPSC. This is not ideal as these are different transgenic lines to begin with and the D2R-cre is a BAC line and the D1R-Cre line is seemingly not. Overall there can be different efficiencies in cre expression between the lines. Also the topographical distribution of VP-projecting D1R and D2R cells in the NAc may be quite different. Overall it is therefore unclear whether similar numbers of NAc cells have ended up being infected in the two lines with the viral approach, complicating such comparisons. In my view the authors should either/or:

- Provide some evidence for (generally) similar levels of opsin in NAc in D1R and D2R-cre lines.

- Swap the current Figure 1 and Figure 2 approach (which have a VP cellular subtype focus, leading to comparisons between transgenic lines), for the Suppl Fig 1 approach (which has a MSN subtype output focus, leading to a more comparable ‘comparison within transgenic line’).

3. Authors argue that it’s reasonable to refer to GAD negative or Vglut2 negative cells as being glutamatergic or GABAergic, respectively. I do not agree that this terminology is wise, especially since, as the authors indicate, there are some other neuronal populations in VP, and there is also potential for undetected fluorescence. Whenever authors identify a cell type based on the absence of fluorescence, they should consistently refer to its identity as putative (or as GAD- or Vglut2-).

4. The approach to evaluate the effect of neuropeptide wash in on both EPSCs and IPSCs in the same cell is in principle elegant though also has a drawback. The baseline for EPSCs is remote from the evaluation of the peptide’s effect on them, and the cell has also been held at 0 mV for 15 minutes in between. This long depolarization in itself may have affected EPSC amplitudes via numerous mechanisms. To attribute effects to the neuropeptides on EPSCs, some control is therefore needed (e.g. same manipulations without wash in of ligand or use of a neuropeptide antagonist). This is particularly needed for the Vglut+ neurons (Figure 3), as for that experiment both peptides are reported to lower EPSC amplitudes. Instead for Vglut2- neurons (Figure 4), the EPSC reduction does not occur with the NK1R ligand, which can serve as a control for the procedure for that particular VP cellular population.

5. Several relevant (methodological) details are absent.

- Age animals

- Precise code of Jax strains used

- Titer and origin of AAV opsin

- Orientation of slicing

- Day-Light cycle rhythm and time(s) of slice preparation with respect to that cycle.

- Duration of the opto pulses.

- In the figures with opto-stimulation, traces are drawn, but the opto pulses are not shown in relation to those examples. This is relevant to at least consider the likelihood of the connections being monosynaptic.

- Not much is said about how the data were analyzed in between acquisition and statistics. What packages/approaches were used?

- Though performed with cesium internals, some of the maximal currents are so large (e.g. exceeding several nanoamperes) that a statement confirming that these are not action currents would be helpful.

- For Figure 5-6, kindly add basal resting membrane resistance and cell capacitance values for these different cell types (baseline). Also report effect of peptide wash in on the membrane resistance.

-

6. Certain textual segments should be enhanced in clarity:

a. The description of the hypothesis should be improved. In the Discussion the authors indicate that expectations were not met, which touches base with their previous rabies study, but a real hypothesis was not made explicit in the introduction.

b. Authors mention that peptide co-release from MSNs can be in the form of substance P, dynorphin, enkephalin and neurotensin. Then subsequently focus solely on NK1R and MOR, but without much explanation on why these were the preferred receptors (given the various options) to then further investigate.

c. Discussion does well to point out certain limitations of the study. The possibility of rabies tropism should be discussed in relation to their previous study.

d. The title is in my view not so accurate as ‘...allow gating of...[...] cells’ is at best missing a noun to indicate what is being gated for those cells (and whether gating is really the appropriate term given the type of measures is arguable).

e. Throughout manuscript (and title) GABAA transmission should be GABAAR or GABAA receptor transmission.

f. In Discussion a limitation to mention is that studies were performed in absence of polysynaptic blocking cocktail TTX/4-AP. Should be discussed in light of likelihood of having picked up polysynaptic effects in Fig 1-4.

g. Generally well written, but a few typos. It can do with another read-through.

--

Reviewer #2

The authors of this work investigated the neurotransmission properties from MSNs in the striatum to neuronal subtypes in the ventral pallidus. Using a combination of genetics, and optogenetic-based functional mapping the authors described that GABA neurons in VP receive similar functional innervation from D1 and D2 MSNs. Glutamate neurons of the VP instead receive larger GABA inputs from the D2 projection rather than the D1 innervation. Furthermore, the analysis of the Mu opioid receptor pathway and the NK1R signaling present independent properties onto GABA and Glutamate cells of the VP.

This work is well presented and conceived. The description of the experiments is nice, and the methodology used is perfect to assess the scientific question. I have very minor points the authors should address:

1-An input output curve using intensity of light is somewhat difficult to assess as the extent of recombination varies across slices, and injected mice. To corroborate these results the authors could also present an analysis of the variance of Cheta expression across slices in order to assess whether the presynaptic innervation was at least comparable.

The experimental conditions enable the authors to study inward GABA currents. Is it possible that variating the Cl- equilibrium also affects somehow the strength of these inputs. The authors could at least run few experiments with GABA currents being outward to assess whether their results are comparable.

In the graphs it is missing when light stimulation occurs to evoke synaptic currents.

2-The recordings using DAMGO and the GR compound are interesting. In this case the authors did not look into D1 or D2 projections specifically, which is a pity for the rest of the study. The authors could consider doing some experiments in this regard.

3- Showing single cell plots is elegant, yet a time course for the effect of the compound on GABA and AMPA current should be in principle performed independently. The possibility the after 10-15 minutes the AMPA current may change because of cell instability or changes in holding current is likely. In addition, this will allow to obtain proper baselines in terms of length and stability of recordings.

4-To corroborate their study the authors could complement their recordings of AMPA currents with that of NMDA receptors, and provide information on all components.

## Author Response

We thank all three reviewers for their thorough and fair reviews.

Reviewer #1

The current study uses path clamp electrophysiological recordings in mouse brain slices, together with optogenetics and pharmacology, to evaluate the connectivity between two separate populations of nucleus accumbens medium spiny neurons (D1R and D2R subtypes) with two subtypes of ventral pallidal neurons (GABA and glutamate). Aside from ionotropic GABAAR mediated transmission in this circuitry, the authors evaluate the effects that two relevant GPCRs (i.e. GPCRs for some of the peptides NAc neurons can corelease) may have in regulating VP cellular subtypes. The authors find that D1R NAc neurons preferentially synapse onto VP GABA over VP putative glutamate neurons. Instead D2R NAc neurons do not have such a preferential innervation pattern. Mu-opioid receptor activation reduces both GABA and glutamate input onto both VP neuron subtypes, and hyperpolarizes VP GABA cells. The effects of NK1R activation was more limited. Overall the study offers some nice further insight in NAc-VP connectivity through a substantial dataset. Some matters do however need to still be addressed:

1. There is generally no histology shown for the experiments. The injections are said to be aimed at the NAc core (at a given set of coordinates), but it is unclear whether they also in practice ended up being selective to the core. It is important to show representative images of this per experiment type (e.g. per different mouse strain injected), as also different mouse lines can have differences in brain anatomy to the point that the same coordinates would end up differently targeting NAc sub-territories across experiments.

We have now added a representative example of viral injection into the nucleus accumbens as a supplementary Figure. While our injections were aimed at the nucleus accumbens core, some spread into the nucleus accumbens shell cannot be excluded. Differences in the brain size or brain anatomy in our mouse lines were not observed.

2. The authors make comparisons across transgenic genotypes. For instance, comparing connectivity of NAc D1 and D2 MSNs with VP neuronal subtypes in such features as maximal IPSC. This is not ideal as these are different transgenic lines to begin with and the D2R-cre is a BAC line and the D1R-Cre line is seemingly not. Overall there can be different efficiencies in cre expression between the lines. Also the topographical distribution of VP-projecting D1R and D2R cells in the NAc may be quite different. Overall it is therefore unclear whether similar numbers of NAc cells have ended up being infected in the two lines with the viral approach, complicating such comparisons. In my view the authors should either/or:

- Provide some evidence for (generally) similar levels of opsin in NAc in D1R and D2R-cre lines.

- Swap the current Figure 1 and Figure 2 approach (which have a VP cellular subtype focus, leading to comparisons between transgenic lines), for the Suppl Fig 1 approach (which has a MSN subtype output focus, leading to a more comparable ‘comparison within transgenic line’).

We have now rearranged and re-analyzed Figures 1 and 2 to allow for a comparison between cell types within the genotype. This should address all concerns regarding differences in viral injection or viral expression between mouse lines.

3. Authors argue that it’s reasonable to refer to GAD negative or Vglut2 negative cells as being glutamatergic or GABAergic, respectively. I do not agree that this terminology is wise, especially since, as the authors indicate, there are some other neuronal populations in VP, and there is also potential for undetected fluorescence. Whenever authors identify a cell type based on the absence of fluorescence, they should consistently refer to its identity as putative (or as GAD- or Vglut2-).

We have now define these terms in the introduction and use GAD2(-/+) or vGlut2(-/+) we are referring to our experimental data and VPGlutamate or VPGABA when providing general discussion of the VP neuronal subtypes.

4. The approach to evaluating the effect of neuropeptide wash-in on both EPSCs and IPSCs in the same cell is in principle elegant though also has a drawback. The baseline for EPSCs is remote from the evaluation of the peptide’s effect on them, and the cell has also been held at 0 mV for 15 minutes in between. This long depolarization in itself may have affected EPSC amplitudes via numerous mechanisms. To attribute effects to the neuropeptides on EPSCs, some control is therefore needed (e.g. same manipulations without wash-in of ligand or use of a neuropeptide antagonist). This is particularly needed for the Vglut+ neurons (Figure 3), as for that experiment both peptides are reported to lower EPSC amplitudes. Instead for Vglut2- neurons (Figure 4), the EPSC reduction does not occur with the NK1R ligand, which can serve as a control for the procedure for that particular VP cellular population.

We have now added control experiments in which cells were recorded at -65 mV, then depolarized to 0 mV, and repolarized again (as in the above-mentioned experiments) without agonist application. These experiments demonstrate that depolarization alone is not significantly changing EPSC amplitude.

5. Several relevant (methodological) details are absent.

- Age animals P60-P90 updated

- Precise code of Jax strains used: updated

- Titer and origin of AAV opsin: Adgene, 7×10¹² vg/ml; updated

- Orientation of slicing; coronal slices, updated

- Day-Light cycle rhythm and time(s) of slice preparation with respect to that cycle. 12h/12h recordings during dark cycle, updated.

- Duration of the opto pulses. 3ms, updated.

- In the figures with opto-stimulation, traces are drawn, but the opto pulses are not shown in relation to those examples. This is relevant to at least consider the likelihood of the connections being monosynaptic. Updated

- Not much is said about how the data were analyzed in between acquisition and statistics. What packages/approaches were used? We have analyzed our raw data in Axograph. This is now added to the Material and Methods section.

- Though performed with cesium internals, some of the maximal currents are so large (e.g. exceeding several nanoamperes) that a statement confirming that these are not action currents would be helpful. We used a voltage-gated sodium channel blocker in the internal (QX-314) in our internal, which prevents the generation of APs, even in the event of poor space clamp. We have previously not mentioned this in the CSCL internal ingredients list but have now updated this accordingly.

- For Figure 5-6, kindly add basal resting membrane resistance and cell capacitance values for these different cell types (baseline). Also report effect of peptide wash in on the membrane resistance. We have now added a supplementary figure showing the input resistance at baseline and after peptide wash in.

6. Certain textual segments should be enhanced in clarity:

a. The description of the hypothesis should be improved. In the Discussion the authors indicate that expectations were not met, which touches base with their previous rabies study, but a real hypothesis was not made explicit in the introduction.

The Introduction now specifically states two hypotheses that were evaluated as follows:

‘Here we used circuit-specific optogenetics in transgenic reporter mouse lines combined with whole-cell patch-clamp recordings to characterize the functional connectivity between the D1/D2-MSNs and VPGABA/VPGAD2(-) neurons and test an hypothesis that the apparent bias in anatomical connectivity (Heinsbroek et al., 2020) is recapitulated in biased functional connectivity. We furthermore used MOR and NK1R agonists to evaluate the potential cell type-specific modulation by two of the co-released neuropeptides.’

b. Authors mention that peptide co-release from MSNs can be in the form of substance P, dynorphin, enkephalin and neurotensin. Then subsequently focus solely on NK1R and MOR, but without much explanation on why these were the preferred receptors (given the various options) to then further investigate.

We provide the following statement in the introduction regarding our choice of which peptides to evaluate:

‘Given the previous literature that stimulation of MOR or NK1R in the VP can promote motivated behavior we hypothesized that agonists at these receptors would either stimulate VPGAD2(+)or inhibit VPGAD2(-).’

c. Discussion does well to point out certain limitations of the study. The possibility of rabies tropism should be discussed in relation to their previous study.

We added this to our discussion.

d. The title is in my view not so accurate as ‘...allow gating of...[...] cells’ is at best missing a noun to indicate what is being gated for those cells (and whether gating is really the appropriate term given the type of measures is arguable).

We appreciate this comment and the title has been changed to:

“;Differential modulation of GABAergic and glutamatergic neurons in the ventral pallidum by GABA and neuropeptide”

e. Throughout manuscript (and title) GABAA transmission should be GABAAR or GABAA receptor transmission.

Thank you, this change has been made.

f. In Discussion a limitation to mention is that studies were performed in absence of polysynaptic blocking cocktail TTX/4-AP. Should be discussed in light of likelihood of having picked up polysynaptic effects in Fig 1-4.

We have added this possibility to our limitations section.

g. Generally well written, but a few typos. It can do with another read-through.

--

Reviewer #2

The authors of this work investigated the neurotransmission properties from MSNs in the striatum to neuronal subtypes in the ventral pallidus. Using a combination of genetics, and optogenetic-based functional mapping the authors described that GABA neurons in VP receive similar functional innervation from D1 and D2 MSNs. Glutamate neurons of the VP instead receive larger GABA inputs from the D2 projection rather than the D1 innervation. Furthermore, the analysis of the Mu opioid receptor pathway and the NK1R signaling present independent properties onto GABA and Glutamate cells of the VP.

This work is well presented and conceived. The description of the experiments is nice, and the methodology used is perfect to assess the scientific question. I have very minor points the authors should address:

1-An input output curve using intensity of light is somewhat difficult to assess as the extent of recombination varies across slices, and injected mice. To corroborate these results the authors could also present an analysis of the variance of Cheta expression across slices in order to assess whether the presynaptic innervation was at least comparable.

We have now rearranged and re-analyzed Figures 1 and 2 to allow for a comparison between cell types within genotype. This should address all concerns regarding differences in viral injection or viral expression between mouse lines.

The experimental conditions enable the authors to study inward GABA currents. Is it possible that variating the Cl- equilibrium also affects somehow the strength of these inputs. The authors could at least run few experiments with GABA currents being outward to assess whether their results are comparable.

Please note that for the experiments in Figure 1+2 we used a high Chloride internal solution. Hence we are measuring the outward flow of Cl-.

2-The recordings using DAMGO and the GR compound are interesting. In this case the authors did not look into D1 or D2 projections specifically, which is a pity for the rest of the study. The authors could consider doing some experiments in this regard.

These would be highly interesting experiments, unfortunately they would take many months of work, so we are unable to perform them for this manuscript. We will however make sure to discuss the implications of not differentiating between D1R and D2R inputs more thoroughly.

3- Showing single cell plots is elegant, yet a time course for the effect of the compound on GABA and AMPA current should be in principle performed independently. The possibility the after 10-15 minutes the AMPA current may change because of cell instability or changes in holding current is likely. In addition, this will allow to obtain proper baselines in terms of length and stability of recordings.

We have now added control experiments in which cells were recorded at -65 mV, than depolarized to 0 mV and repolarized again (as in the above mentioned experiments) without agonist application. These experiments demonstrate that depolarization alone is not significantly changing EPSC amplitude.

## References

[B1] Bengtson CP, Osborne PB (2000) Electrophysiological properties of cholinergic and noncholinergic neurons in the ventral pallidal region of the nucleus basalis in rat brain slices. J Neurophysiol 83:2649–2660. 10.1152/jn.2000.83.5.2649 10805665

[B2] Cifuentes F, Morales MA (2021) Functional implications of neurotransmitter segregation. Front Neural Circuits 15:738516. 10.3389/fncir.2021.738516 34720888 PMC8548464

[B3] Creed M, Ntamati NR, Chandra R, Lobo MK, Lüscher C (2016) Convergence of reinforcing and anhedonic cocaine effects in the ventral pallidum. Neuron 92:214–226. 10.1016/j.neuron.2016.09.001 27667004 PMC8480039

[B4] Faget L, Zell V, Souter E, McPherson A, Ressler R, Gutierrez-Reed N, Yoo JH, Dulcis D, Hnasko TS (2018) Opponent control of behavioral reinforcement by inhibitory and excitatory projections from the ventral pallidum. Nat Commun 9:849. 10.1038/s41467-018-03125-y 29487284 PMC5829073

[B5] Francis TC, Yano H, Demarest TG, Shen H, Bonci A (2019) High-frequency activation of nucleus accumbens D1-MSNs drives excitatory potentiation on D2-MSNs. Neuron 103:432–444.e3. 10.1016/j.neuron.2019.05.031 31221559 PMC6712577

[B6] Hasenöhrl RU, Souza-Silva MAD, Nikolaus S, Tomaz C, Brandao ML, Schwarting RKW, Huston JP (2000) Substance P and its role in neural mechanisms governing learning, anxiety and functional recovery. Neuropeptides 34:272–280. 10.1054/npep.2000.0824 11049731

[B7] Heinsbroek JA, Neuhofer D, Griffin WC, Siegel GS, Bobadilla A, Kupchik YM, Kalivas PW (2017) Loss of plasticity in the D2-accumbens pallidal pathway promotes cocaine seeking. J Neurosci 37:757–767. 10.1523/JNEUROSCI.2659-16.2016 28123013 PMC5296778

[B8] Heinsbroek JA, Bobadilla A, Dereschewitz E, Assali A, Chalhoub RM, Cowan CW, Kalivas PW (2020) Opposing regulation of cocaine seeking by glutamate and GABA neurons in the ventral pallidum article opposing regulation of cocaine seeking by glutamate and GABA neurons in the ventral pallidum. Cell Rep 30:2018–2027.e3. 10.1016/j.celrep.2020.01.023 32049028 PMC7045305

[B9] Jullié D, Valbret Z, Stoeber M (2022) Optical tools to study the subcellular organization of GPCR neuromodulation. J Neurosci Methods 366:109408. 10.1016/j.jneumeth.2021.109408 34763022

[B10] Kalivas PW, Churchill L, Romanides A (1999) Involvement of the pallidal-thalamocortical circuit in adaptive behavior. Ann N Y Acad Sci 877:64–70. 10.1111/j.1749-6632.1999.tb09261.x 10415643

[B11] Knowland D, Lilascharoen V, Pacia CP, Shin S, Wang EH, Lim BK (2017) Distinct ventral pallidal neural populations mediate separate symptoms of depression. Cell 170:284–297.e18. 10.1016/j.cell.2017.06.015 28689640 PMC5621481

[B12] Kupchik YM, Scofield MD, Rice KC, Cheng K, Roques BP, Kalivas PW (2014) Cocaine dysregulates opioid gating of GABA neurotransmission in the ventral pallidum. J Neurosci 34:1057–1066. 10.1523/JNEUROSCI.4336-13.2014 24431463 PMC3891949

[B13] Kupchik YM, Brown RM, Heinsbroek JA, Lobo MK, Schwartz DJ, Kalivas PW (2015) Coding the direct/indirect pathways by D1 and D2 receptors is not valid for accumbens projections. Nat Neurosci 18:1230–1232. 10.1038/nn.4068 26214370 PMC4551610

[B14] Mahler SV, Vazey EM, Beckley JT, Keistler CR, McGlinchey EM, Kaufling J, Wilson SP, Deisseroth K, Woodward JJ, Aston-Jones G (2014) Designer receptors show role for ventral pallidum input to ventral tegmental area in cocaine seeking. Nat Neurosci 17:577–585. 10.1038/nn.3664 24584054 PMC3973180

[B15] Margeta-Mitrovic M, Mitrovic I, Riley RC, Jan LY, Basbaum AI (1999) Immunohistochemical localization of GABA(B) receptors in the rat central nervous system. J Comp Neurol 405:299–321. 10.1002/(SICI)1096-9861(19990315)405:3<299::AID-CNE2>3.0.CO;2-6 10076927

[B16] Matsumura S, Yamamoto K, Nakaya Y, O’Hashi K, Kaneko K, Takei H, Tsuda H, Shirakawa T, Kobayashi M (2021) Presynaptic NK1 receptor activation by substance P suppresses EPSCs via nitric oxide synthesis in the rat insular cortex. Neuroscience 455:151–164. 10.1016/j.neuroscience.2020.12.012 33359655

[B17] Mengual E, Chan J, Lane D, San Luciano Palenzuela M, Hara Y, Lessard A, Pickel VM (2008) Neurokinin-1 receptors in cholinergic neurons of the rat ventral pallidum have a predominantly dendritic distribution that is affected by apomorphine when combined with startle-evoking auditory stimulation. Neuroscience 151:711–724. 10.1016/j.neuroscience.2007.08.039 18178320 PMC2268874

[B18] North RA, Williams JT, Surprenant A, Christie MJ (1987) Μ and Δ receptors belong to a family of receptors that are coupled to potassium channels. Proc Natl Acad Sci U S A 84:5487–5491. 10.1073/pnas.84.15.5487 2440052 PMC298884

[B19] Ottenheimer D, Richard JM, Janak PH (2018) Ventral pallidum encodes relative reward value earlier and more robustly than nucleus accumbens. Nat Commun 9:4350. 10.1038/s41467-018-06849-z 30341305 PMC6195583

[B20] Ottenheimer DJ, Bari BA, Sutlief E, Fraser KM, Kim TH, Richard JM, Cohen JY, Janak PH (2020) A quantitative reward prediction error signal in the ventral pallidum. Nat Neurosci 23:1267–1276. 10.1038/s41593-020-0688-5 32778791 PMC7870109

[B21] Pardo-Garcia TR, Garcia-Keller C, Penaloza T, Richie CT, Pickel J, Hope BT, Harvey BK, Kalivas PW, Heinsbroek JA (2019) Ventral pallidum is the primary target for accumbens D1 projections driving cocaine seeking. J Neurosci 39:2041–2051. 10.1523/JNEUROSCI.2822-18.2018 30622165 PMC6507080

[B22] Parrilla-Carrero J, Eid M, Li H, Chao YS, Jhou TC (2021) Synaptic adaptations at the rostromedial tegmental nucleus underlie individual differences in cocaine avoidance behavior. J Neurosci 41:4620–4630. 10.1523/JNEUROSCI.1847-20.2021 33753546 PMC8260244

[B23] Richard JM, Ambroggi F, Janak PH, Fields HL, Richard JM, Ambroggi F, Janak PH, Fields HL (2016) Ventral pallidum neurons encode incentive value and promote cue-elicited instrumental actions report ventral pallidum neurons encode incentive value and promote cue-elicited instrumental actions. Neuron 90:1165–1173. 10.1016/j.neuron.2016.04.037 27238868 PMC4911300

[B24] Root DH, Melendez RI, Zaborszky L, Napier TC (2015) The ventral pallidum: subregion-specific functional anatomy and roles in motivated behaviors. Prog Neurobiol 130:29–70. 10.1016/j.pneurobio.2015.03.005 25857550 PMC4687907

[B25] Smith KS, Berridge KC (2005) The ventral pallidum and hedonic reward: neurochemical maps of sucrose “liking” and food intake. J Neurosci 25:8637–8649. 10.1523/JNEUROSCI.1902-05.2005 16177031 PMC6725525

[B26] Smith KS, Tindell AJ, Aldridge JW, Berridge KC (2009) Ventral pallidum roles in reward and motivation. Behav Brain Res 196:155–167. 18955088 10.1016/j.bbr.2008.09.038 PMC2606924

[B27] Smith RJ, Lobo MK, Spencer S, Kalivas PW (2013) Cocaine-induced adaptations in D1 and D2 accumbens projection neurons (a dichotomy not necessarily synonymous with direct and indirect pathways). Curr Opin Neurobiol 23:546–552. 10.1016/j.conb.2013.01.026 23428656 PMC3681928

[B28] Soares-Cunha C, de Vasconcelos NAP, Coimbra B, Domingues AV, Silva JM, Loureiro-Campos E, Gaspar R, Sotiropoulos I, Sousa N, Rodrigues AJ (2020) Nucleus accumbens medium spiny neurons subtypes signal both reward and aversion. Mol Psychiatry 25:3241–3255. 10.1038/s41380-019-0484-3 31462765 PMC7714688

[B29] Stephenson-Jones M, Bravo-Rivera C, Ahrens S, Furlan A, Xiao X, Fernandes-Henriques C, Li B (2020) Opposing contributions of GABAergic and glutamatergic ventral pallidal neurons to motivational behaviors. Neuron 105:921–933.e5. 10.1016/j.neuron.2019.12.006 31948733 PMC8573387

[B30] Tang X-C, McFarland K, Cagle S, Kalivas PW (2005) Cocaine-induced reinstatement requires endogenous stimulation of μ-opioid receptors in the ventral pallidum. J Neurosci 25:4512–4520. 10.1523/JNEUROSCI.0685-05.2005 15872098 PMC6725035

[B31] Tejeda HA, Wu J, Kornspun AR, Pignatelli M, Kashtelyan V, Krashes MJ, Lowell BB, Carlezon WA, Bonci A (2017) Pathway- and cell-specific kappa-opioid receptor modulation of excitation-inhibition balance differentially gates D1 and D2 accumbens neuron activity. Neuron 93:147–163. 10.1016/j.neuron.2016.12.005 28056342 PMC5808882

[B32] Tepper JM, Bolam JP (2004) Functional diversity and specificity of neostriatal interneurons. Curr Opin Neurobiol 14:685–692. 10.1016/j.conb.2004.10.003 15582369

[B33] Tindell AJ, Berridge KC, Zhang J, Peciña S, Aldridge JW (2005) Ventral pallidal neurons code incentive motivation: amplification by mesolimbic sensitization and amphetamine. Eur J Neurosci 22:2617–2634. 10.1111/j.1460-9568.2005.04411.x 16307604

[B34] Tooley J, Marconi L, Alipio JB, Matikainen-ankney B, Georgiou P, Kravitz AV, Creed MC (2018) Glutamatergic ventral pallidal neurons modulate activity of the habenula–tegmental circuitry and constrain reward seeking. Biol Psychiatry 83:1012–1023. 10.1016/j.biopsych.2018.01.003 29452828 PMC5972062

[B35] Torregrossa M, Kalivas PW (2008) Neurotensin in the ventral pallidum increases extracellular γ-aminobutyric acid and differentially affects cue-and cocaine-primed reinstatement. J Pharmacol Exp Ther 325:556–566. 10.1124/jpet.107.130310 18252810 PMC2672956

[B36] Vachez YM, Tooley JR, Abiraman K, Matikainen-Ankney B, Casey E, Earnest T, Ramos LM, Silberberg H, Godynyuk E, Uddin O, Marconi L, Le Pichon CE, Creed MC (2021) Ventral arkypallidal neurons inhibit accumbal firing to promote reward consumption. Nat Neurosci 24:379–390. 10.1038/s41593-020-00772-7 33495635 PMC7933121

[B37] Vanderah T, Sandweiss A (2015) The pharmacology of neurokinin receptors in addiction: prospects for therapy. Subst Abuse Rehabil 6:93–102. 10.2147/SAR.S70350 26379454 PMC4567173

