# Supplementary figures and images for: Differential Modulation of GABAergic and Glutamatergic Neurons in the Ventral Pallidum by GABA and Neuropeptides
Source: eNeuro. 2023 Jul 10;10(7):ENEURO.0404-22.2023. doi: 10.1523/ENEURO.0404-22.2023 (PMC10348443; doi:10.1523/ENEURO.0404-22.2023)

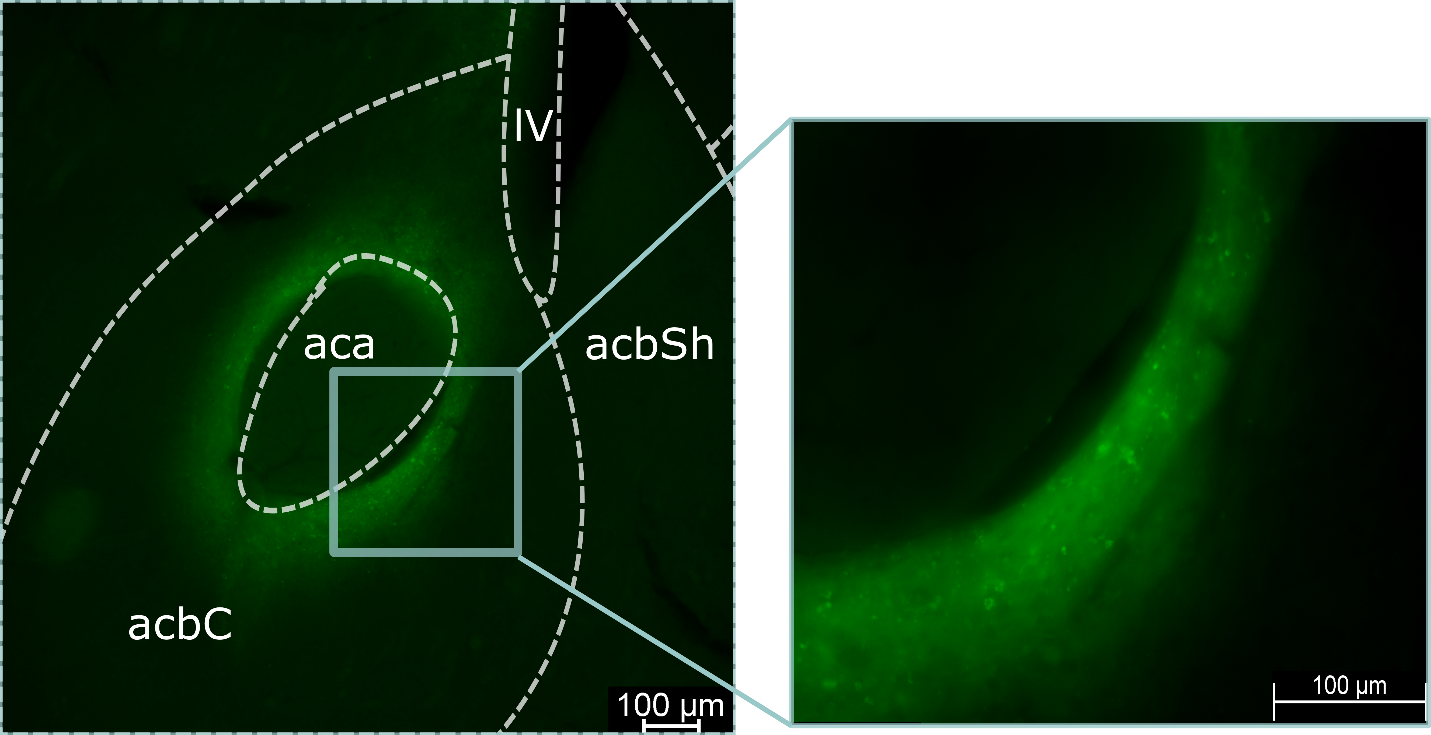

Supplement: Figure 1-1 — Representative image of viral expression in the nucleus accumbens core of a GAD2xD1-cre mouse killed 4 weeks after injection of 280 nl of AAV5-Ef1a-DIO ChETA-EYFP. Aca, Anterior commissure; lV, lateral ventricle; acbC, nucleus accumbens core; acbSh, nucleus accumbens shell. Download Figure 1-1, DOCX file. [file enu-eN-NWR-0404-22-s02.docx]
