# Supplementary material for: Differential Modulation of GABAergic and Glutamatergic Neurons in the Ventral Pallidum by GABA and Neuropeptides
Source: eNeuro. 2023 Jul 10;10(7):ENEURO.0404-22.2023. doi: 10.1523/ENEURO.0404-22.2023 (PMC10348443; doi:10.1523/ENEURO.0404-22.2023)
Supplement: Table 1-1 — Kolmogorov–Smirnov normality tests. Download Table 1-1, DOCX file. [file enu-eN-NWR-0404-22-s05.docx]

**Table 1-1 Kolmogorov-Smirnov normality tests**

| Figure 1 C right: max. response | **GAD2(+)** | | | **GAD2(-)** | | |  |  |  |  |  |  |
| --- | --- | --- | --- | --- | --- | --- | --- | --- | --- | --- | --- | --- |
|  | **N** | **KS** | **p** | **N** | **KS** | **p** |  |  |  |  |  |  |
|  | 23 | 0.26 | <0.0-1 | 14 | 0.23 | <0.05 |  |  |  |  |  |  |
| Figure 1D right: PP-Ratio | **GAD2(+)** | | | **GAD2(-)** | | |  |  |  |  |  |  |
|  | **N** | **KS** | **p** | **N** | **KS** | **p** |  |  |  |  |  |  |
|  | 24 | 0.13 | >0.1 | 13 | 0.11 | >0.1 |  |  |  |  |  |  |
| Figure 2 C right: max. response | **GAD2(+)** | | | **GAD2(-)** | | |  |  |  |  |  |  |
|  | **N** | **KS** | **p** | **N** | **KS** | **p** |  |  |  |  |  |  |
|  | 24 | 0.29 | >0.01 | 13 | 0.28 | >01 |  |  |  |  |  |  |
| Figure 2D right: PP-Ratio | **GAD2(+)** | | | **GAD2(-)** | | |  |  |  |  |  |  |
|  | **N** | **KS** | **p** | **N** | **KS** | **p** |  |  |  |  |  |  |
|  | 24 | 0.16 | 0.09 | 12 | 0.13 | >0.1 |  |  |  |  |  |  |
| Figure 3C left: DAMGO Inhibition | **IPSC** | | | **EPSC** | | |  |  |  |  |  |  |
|  | **N** | **KS** | **p** | **N** | **KS** | **p** |  |  |  |  |  |  |
|  | 6 | 0.18 | >0.1 | 6 | 0.28 | >0.1 |  |  |  |  |  |  |
| Figure 3C right: PP-ratio DAMGO | **IPSC pre** | | | **IPSC post** | | | **EPSC pre** | | | **EPSC post** | | |
|  | **N** | **KS** | **P** | **N** | **KS** | **p** | **N** | **KS** | **p** | **N** | **KS** | **p** |
|  | 6 | 0.3 | >0.1 | 6 | 0.17 | >0.1 | 6 | 0.14 | >0.1 | 6 | 0.22 | >0.1 |
| Figure 3D left: GR73632 Inhibition | **IPSC pre** | | | **IPSC post** | | |  |  |  |  |  |  |
|  | **N** | **KS** | **P** | **N** | **KS** | **p** |  |  |  |  |  |  |
|  | 6 | 0.25 | 0.08 | 6 | 0.21 | >0.1 |  |  |  |  |  |  |
| Figure 3D right: PP-ratio GR73632 | **IPSC pre** | | | **IPSC post** | | | **EPSC pre** | | | **EPSC post** | | |
|  | **N** | **KS** | **P** | **N** | **KS** | **p** | **N** | **KS** | **p** | **N** | **KS** | **p** |
|  | 9 | 0.25 | >0.1 | 9 | 0.27 | 0.06 | 9 | 0.22 | >0.1 | 9 | 0.17 | >0.1 |
| Figure 3E left: E/I change | **DAMGO** | | | **GR73632** | | |  |  |  |  |  |  |
|  | **N** | **KS** | **p** | **N** | **KS** | **p** |  |  |  |  |  |  |
|  | 6 | 0.33 | 0.05 | 9 | 0.19 | >0.1 |  |  |  |  |  |  |
| Figure 4C left: DAMGO Inhibition | **IPSC** | | | **EPSC** | | |  |  |  |  |  |  |
|  | **N** | **KS** | **p** | **N** | **KS** | **p** |  |  |  |  |  |  |
|  | 8 | 0.28 | 0.06 | 8 | 0.16 | >0.1 |  |  |  |  |  |  |
| Figure 4C right: PP-ratio DAMGO | **IPSC pre** | | | **IPSC post** | | | **EPSC pre** | | | **EPSC post** | | |
|  | **N** | **KS** | **P** | **N** | **KS** | **p** | **N** | **KS** | **p** | **N** | **KS** | **p** |
|  | 8 | 0.2 | >0.1 | 8 | 0.19 | >0.1 | 8 | 0.27 | >0.1 | 8 | 0.29 | <0.05 |
| Figure 4D left: GR73632 Inhibition | **IPSC pre** | | | **IPSC post** | | |  |  |  |  |  |  |
|  | **N** | **KS** | **P** | **N** | **KS** | **p** |  |  |  |  |  |  |
|  | 7 | 0.17 | >0.1 | 7 | 0.17 | >0.1 |  |  |  |  |  |  |
| Figure 4D right: PP-ratio GR73632 | **IPSC pre** | | | **IPSC post** | | | **EPSC pre** | | | **EPSC post** | | |
|  | **N** | **KS** | **P** | **N** | **KS** | **p** | **N** | **KS** | **p** | **N** | **KS** | **p** |
|  | 5 | 0.23 | >0.1 | 5 | 0.29 | >0.1 | 5 | 0.24 | >0.1 | 5 | 0.35 | <0.05 |
| Figure 4E left: E/I change | **DAMGO** | | | **GR73632** | | |  |  |  |  |  |  |
|  | **N** | **KS** | **p** | **N** | **KS** | **p** |  |  |  |  |  |  |
|  | 7 | 0.28 | >0.1 | 7 | 0.16 | >0.1 |  |  |  |  |  |  |
| Figure 5D left: RMP DAMGO | **pre** | | | **post** | | |  |  |  |  |  |  |
|  | **N** | **KS** | **p** | **N** | **KS** | **p** |  |  |  |  |  |  |
|  | 8 | 0.29 | <0.05 | 8 | 0.31 | <0.05 |  |  |  |  |  |  |
| Figure 5D right: RMP GR73632 | **pre** | | | **post** | | |  |  |  |  |  |  |
|  | **N** | **KS** | **p** | **N** | **KS** | **p** |  |  |  |  |  |  |
|  | 6 | 0.49 | <0.01 | 6 | 0.25 | >0.1 |  |  |  |  |  |  |
| Figure 5F left: firing DAMGO | **pre** | | | **post** | | |  |  |  |  |  |  |
|  | **N** | **KS** | **p** | **N** | **KS** | **p** |  |  |  |  |  |  |
|  | 8 | 0.23 | >0.1 | 8 | 0.24 | >0.1 |  |  |  |  |  |  |
| Figure 5F right: firing GR73632 | **pre** | | | **post** | | |  |  |  |  |  |  |
|  | **N** | **KS** | **p** | **N** | **KS** | **p** |  |  |  |  |  |  |
|  | 6 | 0.40 | <0.05 | 6 | 0.25 | >0.1 |  |  |  |  |  |  |
| Figure 6D left: RMP DAMGO | **pre** | | | **post** | | |  |  |  |  |  |  |
|  | **N** | **KS** | **p** | **N** | **KS** | **p** |  |  |  |  |  |  |
|  | 10 | 0.18 | >0.1 | 10 | 0.18 | >0.1 |  |  |  |  |  |  |
| Figure 6D right: RMP GR73632 | **pre** | | | **post** | | |  |  |  |  |  |  |
|  | **N** | **KS** | **p** | **N** | **KS** | **p** |  |  |  |  |  |  |
|  | 8 | 0.21 | >0.1 | 8 | 0.21 | >0.1 |  |  |  |  |  |  |
| Figure 6F left: firing DAMGO | **pre** | | | **post** | | |  |  |  |  |  |  |
|  | **N** | **KS** | **p** | **N** | **KS** | **p** |  |  |  |  |  |  |
|  | 10 | 0.15 | >0.1 | 10 | 0.26 | >0.05 |  |  |  |  |  |  |
| Figure 6F right: firing GR73632 | **pre** | | | **post** | | |  |  |  |  |  |  |
|  | **N** | **KS** | **p** | **N** | **KS** | **p** |  |  |  |  |  |  |
|  | 8 | 0.23 | >0.1 | 8 | 0.2 | >0.1 |  |  |  |  |  |  |
