# Supplementary material for: Differential Modulation of GABAergic and Glutamatergic Neurons in the Ventral Pallidum by GABA and Neuropeptides
Source: eNeuro. 2023 Jul 10;10(7):ENEURO.0404-22.2023. doi: 10.1523/ENEURO.0404-22.2023 (PMC10348443; doi:10.1523/ENEURO.0404-22.2023)
Supplement: Supplementary file 3 [file enu-eN-NWR-0404-22-s03.docx]

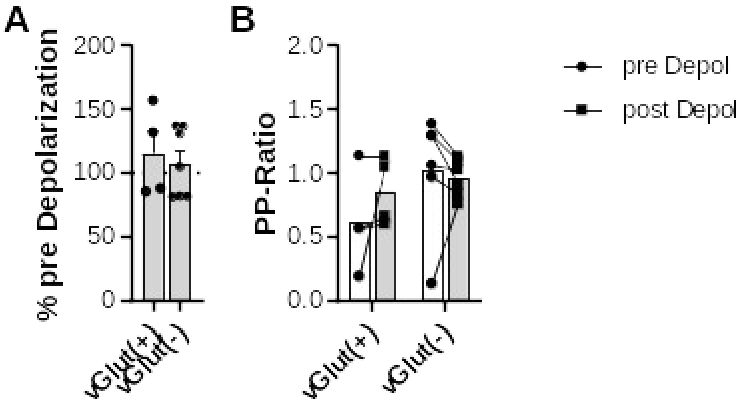

A
% pre Depolarization
200
150
100
50
0
vGlut(+)
vGlut(-)
B
PP-Ratio
2.0
1.5
1.0
0.5
0.0
vGlut(+)
vGlut(-)
pre Depol
post Depol
