# Supplementary material for: Differential Modulation of GABAergic and Glutamatergic Neurons in the Ventral Pallidum by GABA and Neuropeptides
Source: eNeuro. 2023 Jul 10;10(7):ENEURO.0404-22.2023. doi: 10.1523/ENEURO.0404-22.2023 (PMC10348443; doi:10.1523/ENEURO.0404-22.2023)
Supplement: Supplementary file 4 [file enu-eN-NWR-0404-22-s04.docx]

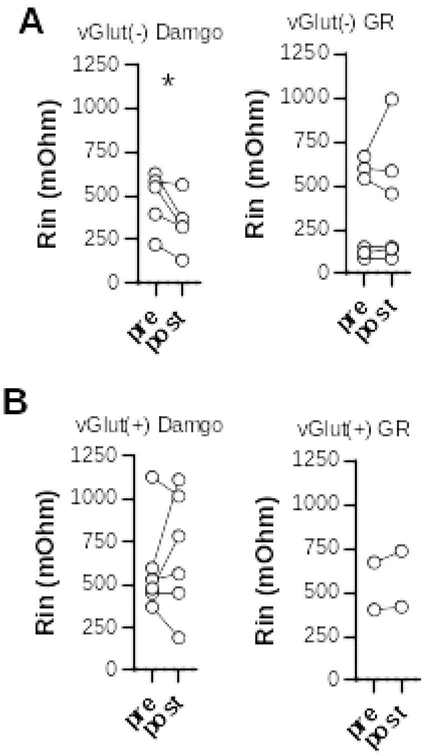

A
vGlut(-) Damgo
vGlut(-) GR
B
vGlut(+) Damgo
vGlut(+) GR
Rin (mOhm)
1250
1000
750
500
250
0
*
pre
post
